# Nutrition, Physical Activity, and Other Lifestyle Factors in the Prevention of Cognitive Decline and Dementia

**DOI:** 10.3390/nu13114080

**Published:** 2021-11-15

**Authors:** Ligia J. Dominguez, Nicola Veronese, Laura Vernuccio, Giuseppina Catanese, Flora Inzerillo, Giuseppe Salemi, Mario Barbagallo

**Affiliations:** 1 Geriatric Unit, Department of Medicine, University of Palermo, 90100 Palermo, Italy; nicola.veronese@unipa.it (N.V.); lvernuccio@virgilio.it (L.V.); giusi.catanese@libero.it (G.C.); florainzerillo@gmail.com (F.I.); mario.barbagallo@unipa.it (M.B.); 2 Faculty of Medicine and Surgery, University of Enna “Kore”, 94100 Enna, Italy; 3 Department of Biomedicine, Neuroscience, and Advanced Diagnostics, University of Palermo, 90100 Palermo, Italy; giuseppe.salemi@unipa.it; 4 UOC of Neurology, University Hospital “Paolo Giaccone”, 90100 Palermo, Italy

**Keywords:** cognitive decline, Alzheimer, dementia, aging, nutrition, physical activity, exercise, diet, sleep, socialization, oxidative stress, inflammation

## Abstract

Multiple factors combined are currently recognized as contributors to cognitive decline. The main independent risk factor for cognitive impairment and dementia is advanced age followed by other determinants such as genetic, socioeconomic, and environmental factors, including nutrition and physical activity. In the next decades, a rise in dementia cases is expected due largely to the aging of the world population. There are no hitherto effective pharmaceutical therapies to treat age-associated cognitive impairment and dementia, which underscores the crucial role of prevention. A relationship among diet, physical activity, and other lifestyle factors with cognitive function has been intensively studied with mounting evidence supporting the role of these determinants in the development of cognitive decline and dementia, which is a chief cause of disability globally. Several dietary patterns, foods, and nutrients have been investigated in this regard, with some encouraging and other disappointing results. This review presents the current evidence for the effects of dietary patterns, dietary components, some supplements, physical activity, sleep patterns, and social engagement on the prevention or delay of the onset of age-related cognitive decline and dementia.

## 1. Introduction

We are living in an aging world. Currently, most humans worldwide may expect to live beyond 60 years [1], while demographic data foresee that half of children alive in 2010 in nations with the highest life expectancies would be centenarians [2]. However, this optimistic scenario can be shadowed by the numerous people that would be affected with age-associated cognitive decline, which is a chief cause of disability worldwide [3,4]. About forty-seven million people have dementia worldwide with an estimated eight million new diagnoses every year. The most common causes of dementia are Alzheimer’s disease (AD), vascular dementia, and mixed forms [3,4,5]. Currently, there is no effective treatment that may significantly modify the course of dementia [5,6]. Brain pathological changes seem to initiate long before clinical manifestations, which mostly occur in old age. This provides a large period of time to implement prevention strategies effectively delaying age-related cognitive decline and dementia, which is a major public health concern. However, there is no solid evidence supporting a role for pharmacological therapies (i.e., anti-inflammatory medications, estrogen/progestin supplementation, antihypertensives, antidiabetics, dementia medications) in the prevention of cognitive decline in persons with preserved cognitive function or with mild cognitive impairment (MCI) according to a systematic review of fifty-one trials [7]. The evidence is not yet solid for cognitive training examined in a systematic review of eleven trials [8] and on exercise in a systematic review including sixteen trials [9]. Conversely, a multidomain intervention including diet, cognitive training, and physical activity reported significant improvements in a range of cognitive outcomes [10].

The interest on dietary and nutritional components as potential modifiable factors for postponing the onset and severity of age-related cognitive function deterioration has grown in recent decades [11]. In fact, unhealthy diet seems to be a key risk factor. This is exemplified by the fact that during Japan’s nutrition transition from the traditional Japanese diet to the Western diet, AD rates rose from 1% in 1985 to 7% in 2008 [12]. The antioxidant and anti-inflammatory actions of various minerals, micronutrients, and vitamins in regard to cognitive function have been studied [13] as well as components of neuronal membranes, e.g., dietary essential fatty acids [14,15]. Several studies on single or multi-component supplements suggest biological plausibility for the cognitive effects of these preparations, but they do not show solid evidence of these effects [16]. Lately, nutrition research has moved from focusing attention only on single nutrients and foods to investigating dietary patterns based on the notion that combinations of foods and nutrients can have synergistic and/or antagonistic effects beyond single components [17].

It has been calculated that about 3% of all dementia cases could be prevented by increasing levels of physical activity [18,19]. Likewise, an increasing number of studies have emerged indicating the importance of physical activity and exercise for the prevention of the pathological process and complications of dementia [20].

In this narrative review, we examine updated evidence of the effects of dietary patterns, dietary components, and supplements on cognitive function decline and dementia. We also review some non-dietary lifestyle factors (i.e., physical activity/exercise, sleep quality, and socialization), which can contribute in association with dietary factors. We included systematic reviews with or without formal meta-analysis of intervention or observational studies dealing with dietary and non-dietary factors as exposure and cognitive and other related parameters as outcomes. We will also briefly review possible mechanisms that may help explain potential benefits.

## 2. Dietary Patterns

Several food combinations in specific dietary patterns have been studied in regard to cognitive decline from MCI to dementia in various contexts with some exhibiting favorable results and others showing no evidence of such effects. Indeed, a multinutrient approach seems to better support outcomes than single nutrient intervention [17]. Although there is no single dietary intervention that has been definitely proven in randomized control trials (RCTs) to effectively prevent cognitive deterioration and dementia, data from epidemiological studies suggest that following a healthy, balanced diet and lifestyle, which has been confirmed to reduce cardiovascular (CV) risk, may also help with preventing or delaying the onset of AD [21,22,23].

### 2.1. Mediterranean Diet (MedDiet)

This dietary pattern that has been traditionally followed for centuries by populations from the Mediterranean countries has been intensely studied in prospective observational studies and trials conducted in Mediterranean and non-Mediterranean countries with different health outcomes. This extensive evidence supports its beneficial action on the prevention of several non-communicable diseases (NCDs), including cognitive decline and dementia [24,25,26].

A systematic review of prospective cohort studies reported that participants with the highest adherence to the MedDiet vs. those with lowest adherence had a 33% lower risk of developing MCI or AD [27]. In the PREDIMED (Prevención con Dieta Mediterranea) trial, among adults (aged 55 to 80 years) at high CV risk, those following a MedDiet supplemented with extra virgin olive oil (EVOO) or nuts had a reduced incidence of CV events, which are a known risk factor for cognitive decline, compared to participants following a low-fat diet over 5 years of follow-up [28]. In sub-analyses of this trial, an improvement in cognitive function with the MedDiet supplemented with either EVOO or nuts vs. a low-fat diet has been reported [29,30]. In prospective longitudinal studies conducted in the USA [31,32,33,34,35] and France [36,37], a higher adherence to a MedDiet was associated with slower cognitive decline and incident AD. Some reviews [24,38] suggested that there is some evidence showing that following a MedDiet is associated with a reduced risk of developing AD, but that still extensive confirmation in different populations and ethnicities is necessary. There are also some conflicting results. For example, an Australian cohort of healthy adults in The Personality and Total Health (PATH) through Life longitudinal study did not find any protection of the MedDiet for cognitive decline [39], while another Australian study, the Australian Imaging, Biomarkers and Lifestyle (AIBL) study [40], reported that patients with MCI or AD had lower adherence to the MedDiet when compared to healthy controls. Tangney et al. evaluated adherence to the Healthy Eating Index (HEI)-2005 or to the MedDiet in regard to cognitive function modifications in 3790 over-65 adults followed for seven years from the Chicago Health and Aging Project longitudinal study. They observed that higher MedDiet adherence scores were associated with slower rates of cognitive decline, after adjusting for confounders, but no association was observed for HEI-2005 scores [41]. Table 1 shows the summary of systematic reviews and meta-analyses exploring the association of adherence to the MedDiet with cognitive decline and/or incident dementia. As mentioned, the MedDiet is the dietary pattern with the highest number of studies published hitherto in the medical literature.

Noteworthy, the MedDiet comprises several other lifestyle parameters, such as physical activity, social engagement, culinary activities, and adequate rest [42], which have shown positive effects on delaying cognitive function deterioration. Therefore, it is crucial to include these other lifestyle factors when evaluating the MedDiet and cognitive performance decline.

**Table 1:** Summary of systematic reviews and meta-analyses exploring the association of adherence to the MedDiet, which is the most studied dietary pattern in the medical literature, with cognitive decline and/or incident dementia.

First Author of the Review	Year	Type of Review	Total Sample Size	N and Type of Studies Included	Summary of Results
Sofi [43]	2008	MA	133,626	2 cohorts (3 studies)	Higher adherence to the MedDiet was associated with a significant reduction in incident PD and AD (RR = 0.87; 95% CI = 0.80, 0.96).
Sofi [24]	2010	MA	136,235	5 prospective cohort studies	Two-point increase in adherence to the MedDiet was associated with a significant reduction of neurodegenerative diseases (RR = 0.87; 95% CI: 0.81, 0.94).
Psaltopoulou [44]	2013	MA	11,141	5 prospective cohort studies; 5 cross-sectional; 2 case control	High and moderate adherence to the MedDiet was associated with reduced risk for cognitive impairment and AD (RR = 0.60, 95% CI = 0.43–0.83).
Singh [27]	2014	SR + MA	7285	5 prospective cohort studies	Highest tertile of the MedDiet adherence was associated with less risk of cognitive impairment (MCI or AD) vs. the lowest tertile. In cognitively normal individuals, higher adherence to the MedDiet was associated with a reduced risk of MCI (HR = 0.73; 95% CI, 0.56–0.96) and AD (HR = 0.64; 95% CI, 0.46–0.89).
Cao [45]	2016	SR + MA	8174	4 prospective cohort studies	MedDiet was significantly associated with reduced incident dementia (RR: 0.69; 95% CI: 0.57, 0.84).
Wu [46]	2017	SR + MA	34,168	9 prospective cohort studies	The highest MedDiet score was inversely associated with the developing of cognitive disorders vs. the lowest category (pooled RR = 0.79, 95% CI: 0.70, 0.90). Median category of adherence was not significantly associated with cognitive disorders. Dose–response analysis indicated a trend of linear association of MedDiet score with the incident risk of cognitive disorders.
Loughrey [47]	2017	SR + MA	41,963	15 prospective cohort studies; 2 RCT	Meta-analysis of cohort studies showed a significant association of MedDiet with episodic memory and global cognition but not working memory or semantic memory. Meta-analysis of RCTs showed that adherence to the MedDiet improved delayed recall, working memory, and global cognition, but not episodic memory, immediate recall, paired associates, attention, processing speed, or verbal fluency vs. controls. The strongest evidence suggests benefit on older adults’ global cognition.
Lourida [48]	2013	SR	18,926	11 cohort studies (8 longitudinal, 2 cross-sectional); 1 RCT	Higher adherence to the Mediterranean diet was associated with better cognitive function, lower rates of cognitive decline, and reduced risk of AD in 9 out of 12 studies, whereas results for MCI were inconsistent.
van de Rest [49]	2015	SR	84,481	6 cross-sectional; 12 longitudinal; 1 trial; 3 meta-analysis	Better adherence to the MedDiet was associated with less cognitive decline, dementia, or AD, as shown by 4 of 6 cross-sectional studies, 6 of 12 longitudinal studies, 1 trial, and 3 meta-analyses.
Petersson [50]	2016	SR	89,561	27 observational studies (16 longitudinal, 6 cross-sectional); 5 RCTs	Most of studies showed an association of adherence to the MedDiet with better cognitive performance. Only 3 of 32 studies found no correlation between MedDiet and AD, 3 found no association between MedDiet and cognitive impairment, and 5 found no association between the MedDiet and cognitive function.
Hardman [51]	2016	SR	59,928	13 prospective cohort studies; 5 RCTs	Higher adherence to the MedDiet was associated with slower cognitive decline, reduced progression to AD, and improvements in cognitive function in the following specific cognitive domains: memory (delayed recognition, long-term, and working memory), executive function, and visual constructs. There are a number of methodological issues.
Aridi [52]	2017	SR	88,419	6 cross-sectional; 23 prospective cohorts; 2 RCTS	Cross-sectional and cohort studies in non-Mediterranean regions showed mixed results. Cohort studies from Mediterranean countries and RCTs showed a beneficial effect of the MedDiet on cognitive function and prevention of AD and dementia.
Knight [53]	2017	SR	44,733	2 cross-sectional; 2 cross-sectional/prospective; 11 prospective	Conflicting results and conclusions regarding the efficacy of the MedDiet for age-related cognitive function. Disparity of neuropsychological assessment methods used appeared to be a plausible contributor to the lack of consensus among study findings.
Radd-Vagenas [54]	2018	SR	1888	5 RCTs (one with 5 studies; total of 9 studies)	Results from 5 RCTS are mostly nonsignificant. Only 12.1% of individual cognitive outcomes significantly favored the MedDiet for cognitive performance. Nevertheless, the significant improvements in cognitive combined domains in PREDIMED, the most robustly designed study, warrant additional research.
Dinu [25]	2018	UR	245,799	5 meta-analyses including 3 to 9 cohort, 3 cross-sectional, 1 case-control (11 analyses)	Out of 11 analyses (9 comparing high vs. low adherence to MedDiet; one for each 2-point increased adherence; one for each 1-point increased adherence), 3 reported convincing evidence of benefit, 2 were highly suggestive of benefit, 3 were suggestive of benefit, 1 reported weak evidence, and only 2 reported no evidence.

AD: Alzheimer’s disease; CI: confidence interval; HR: hazard risk; MA: meta-analysis; MCI: mild cognitive impairment; MedDiet: Mediterranean dietary pattern; PD: Parkinson’s disease; PREDIMED: Prevención con Dieta Mediterranea; RCT: randomized controlled trial; RR: relative risk; SR: Systematic Review; UR: Umbrella Review.

### 2.2. Dietary Approaches to Stop Hypertension (DASH)

This dietary pattern has consistently shown significant lowering of various CV outcomes [55]. Wengreen et al. showed that a higher adherence to both DASH and the MedDiet as well as a greater consumption of whole grains, nuts, and legumes were significantly associated with higher Mini-Mental State Examination (MMSE) scores in a prospective study involving 3831 over-65 participants, who were followed for over eleven years [56]. Participants from the Nurses’ Health Study (*n* = 16,144 women from eleven US states) underwent repeated cognitive testing by telephone at baseline (1995 to 2001), with multiple dietary assessments between 1984 and the first cognitive examination. Greater adherence to long-term DASH score was associated with improvements in global cognition and verbal memory, irrespective of apolipoprotein E epsilon4 (APOE ε4) allele status. However, adherence to the DASH score was not associated with changes in cognitive function over 6 years [57]. Several other studies have shown associations of adherence to DASH with better parameters of cognition [58,59], but there are also null [60,61] or mixed results [57,62]. Nevertheless, DASH can be considered a cardioprotective and neuroprotective dietary pattern.

### 2.3. MedDiet-DASH Intervention for Neurodegenerative Delay (MIND Diet)

Based on the evidence that both MedDiet and DASH were associated with lower incidence of cognitive decline and incident AD and were also cardioprotective, Morris et al. proposed a combination of the most neuroprotective components of MedDiet and DASH in a dietary pattern known as MIND diet [58,59]. These two dietary models provide sources of various antioxidants, B vitamins, polyphenols, polyunsaturated fatty acids (PUFAs), monounsaturated fatty acids (MUFAs), and docosahexaenoic acid, which may help to explain why the dietary patterns resulted in better cognitive health: they combined all these neuroprotective nutrients. The MedDiet, DASH, and MIND diets are primarily plant-based and include vegetables, fruits, nuts and seeds, whole grains, legumes, healthy fats, fish, and poultry that are rich in these nutrients. In addition, the MedDiet, DASH, and MIND diets recommend minimizing the consumption of red meat, sweets, processed foods, and sugar-sweetened beverages that are often associated with Western dietary patterns and with negative health outcomes [23,26,63]. Indeed, the Western dietary pattern has been associated with an increased risk of AD, although the underlying mechanisms are not yet completely clear [64]. In experimental studies, the Western diet enhanced brain inflammation and the production of beta-amyloid protein [65]. Furthermore, the MedDiet, DASH, and MIND diets reduce middle-age CV risk factors that are also risk factors for AD, including vascular disease, hypertension, stroke, diabetes, and obesity [26,66,67,68,69,70]. Specifically, vascular disease risk factors, more so in midlife, are associated with brain accumulation of beta-amyloid [71,72]. It has been estimated that about 40% of dementia cases could be prevented or delayed by focusing on modifiable lifestyle risk factors [18]. Thus, emerging research points to the components of these three healthy dietary patterns, which may have synergistic effects impacting physiological processes and signaling pathways associated with cognitive function and decline [73,74,75]. Thus, the MIND diet combining elements of the MedDiet and DASH has been associated with reduced risk of cognitive impairment and incident AD [59,63]. Assessing the adherence to the MIND diet among 960 participants of the Memory and Aging Project followed for an average of 4.7 years, Morris et al. observed a positive association of following the MIND diet with a slower decline in five single cognitive domains and also in global cognitive performance, after adjusting for confounders [59]. Likewise, there was a reduction in incident AD for participants aged 58 to 98 years with the highest adherence to MIND diet compared to the lowest adherence to this dietary pattern [63]. Taken together, most results available testing the MIND diet have been favorable [59,61,63,76], and only one study had mixed results [77].

In summary, the MIND diet can be considered a neuroprotective dietary pattern. Nevertheless, replications of the positive results with the MIND diet are needed in other populations to confirm their relevance to brain health.

### 2.4. Ketogenic Diet

Ketone bodies, products of fat metabolism, are a source of energy for the brain and are available even when glucose supplies are insufficient (e.g., during severe carbohydrate restriction) or when its metabolism is defective as in AD. Based on evidence that this dietary pattern has been associated with positive results in some forms of drug-resistant epilepsy proposed to be mediated by the action of medium-chain triglycerides (MCT) and generation of ketones as an alternative metabolic substrate for the brain, enhancing mitochondrial function and reducing the expression of inflammatory and apoptotic mediators [78,79], this diet has been proposed as neuroprotective. In the last few years, some investigations have suggested that the ketogenic diet, which is characterized by a high consumption of fat, moderate consumption of protein, and very low carbohydrate composition, may be a useful instrument for the prevention of age-associated cognitive decline. However, most available studies are experimental, and there are not yet enough data from clinical trials to draw definitive conclusions on the prevention and treatment of cognitive decline and AD.

In experimental aged animals, the ketogenic diet improved cognitive performance even under hypoxic conditions without changes in motor performance [80]. Another two experimental studies found that ketogenic diet started at a young age improved cognition and extended longevity [81,82]. Late-life ketogenic diet intervention improved behavior and cognitive tasks that required working memory in rats [83]. A preliminary double-blind placebo-controlled study in twenty patients with MCI or AD reported improvement in AD Assessment Scale-Cognitive subscale (ADAS-cog) scores in APOE ε4-negative patients receiving medium-chain triglycerides, which was not observed in APOE ε4-positive patients; higher ketone concentrations were associated with greater improvement in paragraph recall with MCT treatment compared to placebo among all participants [84]. Another study involving 152 participants reported similar results [85]. A recent preliminary human phase I/II RCT examined the feasibility of using a modified Atkins diet (MAD) to induce ketogenesis in persons with MCI or early AD, and it tested as a primary outcome the effects of this diet on a Memory Composite Score (MCS), the sum of the delayed recall trials for the Hopkins Verbal Learning Test—Revised (HVLT-R) and Brief Visuospatial Memory Test—Revised (BVMT-R). Secondary outcomes for efficacy were changes in the MMSE-2—Expanded Version (EV), Profile of Mood States, bipolar form (POMS-BI), and instrumental activities of daily living (IADL) scores and other clinical outcomes. Twenty-seven participants were eligible. After extensive assessment and education, participants were randomly assigned to either the MAD or the National Institute on Aging (NIA) recommended diet for older people for twelve weeks. Preliminary results showed that nine patients in the MAD arm and five in the NIA arm had completed the trial. Despite extensive training, adherence to both diets was only fair. Among those in the MAD arm who generated at least trace amounts of urinary ketones, there was a large and statistically significant increase in MCS between the baseline and week-6 assessment. MAD participants also reported increased energy between baseline and week-6 assessment. These preliminary results suggest that the generation of even trace ketones might enhance episodic memory and vitality in very early AD [86]. Another RCT involving twenty-one patients with clinically confirmed AD were randomly assigned to a modified ketogenic diet or usual diet supplemented with low-fat healthy-eating guidelines in a crossover trial (two 12-week treatment periods, separated by a 10-week washout period). Participants on the ketogenic diet achieved sustained physiological ketosis measured with blood beta-hydroxybutyrate concentrations. Compared with the usual diet, patients on the ketogenic diet had better scores in the AD Cooperative Study—Activities of Daily Living and Quality of Life in AD (ADCS-ADL) testing; Addenbrookes Cognitive Examination-III also improved but not significantly. Changes in CV risk factors were mostly favorable, while adverse effects were mild [87]. These results encourage the design of larger RCTs in AD patients to confirm the positive effects on daily function and quality of life, which are two factors of great importance to persons and caregivers living with dementia. Even if promising, these are still preliminary results that should be validated by future larger studies.

### 2.5. Nordic Diet

The Nordic diet attempts to reflect the diet consumed in Nordic countries, where the usual eating habits include high consumption of fish, cabbages, apples, and pears, whole grains from oat, berries, root vegetables, barley and rye, low-fat dairy products, potatoes, and rapeseed oil [88]. A study including participants aged fifty-seven to seventy-eight years with normal cognition at baseline reported that better adherence to the Nordic diet was associated with higher scores in global cognition over a 4-year study period after adjustment for demographic and lifestyle factors [89]. A large cohort study involving 2223 dementia-free adults (MMSE scores higher than 27/30) aged over sixty years followed for six years found that moderate to high adherence to the Nordic diet was more closely associated with less cognitive decline than moderate to high adherence to other healthy dietary patterns, i.e., the MedDiet, DASH, and MIND [61]. Subsequent analyses of this cohort study aiming to verify whether an active lifestyle may reinforce the positive effects of the Nordic Prudent Dietary Pattern (NPDP) on cognitive function showed that the association of NPDP with a reduced decline in MMSE scoring became stronger when combined with moderate-to-intense physical, mental, or social activities. Thus, the combination of NPDP and an active lifestyle may result in even better preservation of cognitive function and further decreased risk of cognitive decline [90].

In summary, the results of studies with the Nordic diet are still scarce. Although they do appear promising for people with preserved cognitive function, whether such diet might improve cognition in a population with cognitive decline and in populations different from that of Nordic countries remains to be established.

### 2.6. Vegetarian Diet

It is accepted today that dietary patterns that emphasize plant foods, which are plenty of bioactive compounds but not strictly vegetarian/vegan can exert neuroprotective effects. Likewise, it is recognized that an increased consumption of fruits and vegetables can be favorable to brain health. A recent prospective study involving 27,842 men with a mean age of 51 years in 1986 examined the relation of vegetable and fruit consumption, which was measured with five repeated food frequency questionnaire (FFQ)s collected every 4 years, to future incident subjective cognitive function. Higher intakes of total vegetables, total fruits, and fruit juice 18 to 22 years before were each significantly associated with lower odds of moderate or poor subjective cognitive function [91]. Nevertheless, there is no direct evidence yet to support the benefits of a strict vegetarian or vegan diet in preventing cognitive decline despite evidence for brain-health-promoting effects, mainly in experimental and observational investigations, of several plant foods rich in polyphenols and anti-inflammatory components that may impact neuroinflammation [92,93].

In 1993, Giem et al. published preliminary findings of two cohort sub-studies of the Adventist Health Study examining the association of following a vegetarian diet or a meat-eating diet with the incidence of dementia [94]. One of the studies (*n* = 272 participants) showed that persons who frequently consumed meat had an over two-fold increased risk of becoming demented than vegetarians. The second study (*n* = 2984 participants) reported no significant difference in incident dementia in meat-eating participants vs. vegetarians. No explanation for these contrasting results were proposed, and there was no evidence of standardized cognitive assessments during the studies [94]. Although vegetarian/vegan diets may provide beneficial health effects, they may also lead to nutritional deficiencies. A meta-analysis examining the relationship between vegan or vegetarian diets and cognitive and mental health (*n* = 13 articles; 17,809 participants) found no significant association between diet and the continuous depression score, stress, well-being, or cognitive impairment. Vegans/vegetarians were at increased risk for depression and had lower anxiety scores, but no differences for other outcomes, including cognition, were found. Heterogeneity was large, impeding subgroup analyses [95].

### 2.7. Healthy Eating Index-2005 (HEI-2005)

The HEI was developed based on foods aligning the key recommendations of the Dietary Guidelines for Americans in order to measure diet quality. There are conflicting results on the effects of this dietary pattern on cognitive function. Former cross-sectional studies showed associations of HEI-2005 with improvements in cognitive tests [96,97]. Conversely, other longitudinal studies involving participants followed for up to 7.6 years reported no association of HEI-2005 with any modification of cognitive performance [41,98]. Wengreen et al. conducted a longer study with a follow-up of eleven years showing an association between increased HEI scores with lower cognitive decline, suggesting that a longer period of time is needed in order to reveal the effects of diet on cognitive function [99].

### 2.8. Okinawa Diet

This type of diet describes the eating habits followed by people originally from the Ryukyu Island in Japan, which counts the largest number of centenarians in the world. Although this dietary pattern has been associated with a long and healthy life, there is no direct evidence of its effects on the prevention of cognitive deterioration and AD. A not fully reliable study reported a higher incidence of dementia in 157 migrants from Okinawa to Brazil compared to 2217 residents of Okinawa [100]. However, there was a period of nine years between the evaluation of the two groups and a remarkably different sample size, which hindered definitive conclusions.

### 2.9. Low-Copper Diet

A former longitudinal study involving 3718 participants reported that elevated intakes of copper supplements in combination with a diet rich in saturated and trans-fatty acids were associated with faster cognitive decline after a follow-up of five and a half years [101]. Conversely, a small study in thirty-two patients with AD at mild to moderate stages showed that those participants with low plasma concentrations of copper had higher (worse) ADAS-cog scores [102]. It has been suggested that the promotion of a low-copper diet may potentially reduce the risk of AD [103], but there is no evidence that this can modify the incidence or pathological features of AD.

### 2.10. Paleolithic Diet

Although there is no evidence on the prevention of cognitive decline or AD with the Paleolithic diet, some small studies have been conducted in overweight and diabetic patients [104,105]. One of these studies compared the effects of Paleolithic diet vs. the Nordic Nutrition Recommendations in a sample of twenty overweight postmenopausal women followed for six months. The outcomes included parameters of functional magnetic resonance imaging (fMRI), episodic memory fMRI tasks (a face-name paradigm was used to examine brain responses related to episodic memory), and weight loss. The authors reported significant improvements in fMRI episodic memory tests, which were associated with increased hippocampal activity, decreased waist circumference, and reduced plasma free fatty acid with similar results for both dietary patterns [104]. Another study was conducted in twelve sedentary patients with type 2 diabetes (with or without metformin treatment) randomized to follow a Paleolithic diet with or without high-intensity exercise. After twelve weeks, both interventions induced similar significant weight loss, improved insulin sensitivity, and increased oxygen uptake. Both interventions were associated with increased functional brain responses within the right anterior hippocampus and right inferior occipital gyrus as well as increased volume of the right posterior hippocampus, which are areas that are linked to cognitive functions. No changes in episodic memory function and associated functional brain responses were observed in this short period [105]. Further studies are still needed to test the long-term effects of this dietary pattern.

## 3. Dietary Components and Supplements

### 3.1. Vegetables

There is growing evidence that plant-based diets rich in vegetables can be protective, together with other dietary and lifestyle factors, against age-related cognitive decline and can help prevent dementia [92]. In the preceding sections, we have discussed dietary patterns with evidence of cognitive decline and dementia protection. Although those that are most protective, namely the MedDiet, DASH, and MIND [23,26,67,106,107], are not strictly vegetarian, these diets are mainly composed of foods with plant origin, including abundant amounts of vegetables. This means that they are rich in phytonutrients, such as polyphenols, carotenoids, antioxidant vitamins, healthy fats, and other phytochemicals linked to lower chronic inflammation and oxidative stress and to better scores of the Dietary Inflammatory Index, which has been shown to be inversely associated with memory and cognitive functioning [108]. In animal models, these nutrients and non-nutrients have been found to enhance neurogenesis, synaptic plasticity, and neuronal survival by reducing oxidative stress and neuroinflammation [109]. In particular, a variety of vegetables have attracted the attention of the lay public and also the scientific community for their content, although in reduced amounts, of numerous molecules increasingly investigated for their healthy properties. Among them, plant polyphenols have raised notable interest [110]. Various clinical trials have indicated that many health benefits of the Mediterranean and Asian diets can be related to the presence of significant amounts of polyphenols, even if in some cases, inconsistent results have been reported as well, which highlights the need for further investigation.

A recent analysis of data from the Survey of Health, Ageing, and Retirement in Europe (SHARE) (*n* = 22,635) including participants from eleven European countries showed that frequent consumption of fruits and vegetables was associated with improved health outcomes, including cognitive and mental health [111]. In a biracial cohort of 3231 men and women aged 18–30 years at baseline in 1985–1986, which was followed for up to 25 years in the Coronary Artery Risk Development in Young Adults Study conducted in the US, intake of whole vegetables (excluding potatoes) was significantly associated with a better cognitive performance after adjustment for potential confounders in all three cognitive tests used, supporting the long-term benefits of vegetables and fruits consumption on cognitive performance [112]. Among 27,842 men from the Health Professionals Follow-up Study with a mean age of 51 years in 1986 and followed for 18 to 22 years, higher intakes of total vegetables, total fruits, and fruit juice were each significantly associated with lower odds of moderate or poor subjective cognitive function after controlling for major non-dietary factors and total energy intake, confirming the long-term beneficial role of vegetables, fruit, and orange juice on cognition [91]. A systematic review and meta-analysis identified six cohorts involving 21,175 participants. The pooled analysis found that the consumption of fruit and vegetables was inversely associated with the incident risk of cognitive disorders, with evidence of significant heterogeneity attributed to ethnic differences [113]. An observational study examined the diet of 17,700 community-living dementia-free Chinese older adults who attended the Elderly Health Centers in Hong Kong at baseline and followed their cognitive status for six years, evaluating whether the minimal daily requirement of vegetables and fruits recommended by the World Health Organization (WHO) would independently lower dementia risk. Multivariable logistic regression analysis showed that having at least three servings of vegetables and two servings of fruits daily might help prevent dementia in older adults [114]. Another meta-analysis identified nine studies (five cohort studies and four cross-sectional studies) including 31,104 participants and 4583 incident cases of cognitive impairment and dementia. The meta-analysis showed that an increased consumption of fruit and vegetables was associated with a significant reduction in the risk of cognitive impairment and dementia, particularly in men and women aged over 65 years. Dose–response meta-analysis showed that an increment of 100 g per day of fruit and vegetable consumption was related to a near 13% reduction in cognitive impairment and dementia risk [115].

In conclusion, a frequent and abundant consumption of vegetables, possibly within a dietary and lifestyle pattern with other neuroprotective components, appears to positively impact cognitive well-being and help prevent cognitive decline.

### 3.2. Extra-Virgin Olive Oil (EVOO)

Olive oil, a staple of the MedDiet, is a natural product rich in oleic acid, which is a MUFA. Olive oil is the typical culinary fat used in Mediterranean countries where it is the main source of fat in this dietary pattern. There are various types of olive oil (i.e., EVOO, virgin olive oil, refined olive oil, and pomace oil), which exhibit diverse chemical characteristics and potential impacts on human health [116]. Unfortunately, olive oil subtypes are often not discriminated in research investigation with possible heterogeneous results due to the diverse effects of various subtypes. In particular, EVOO arises from the first pressing of fresh olives, usually within 24 h after their harvest. It is extracted by non-chemically induced mechanical means at temperatures below 28 °C. Its free fatty acid or acidity level is lower than 0.8%, having optimal taste and odor. Olives contain hydrophilic phenol compounds, including simple phenolic compounds (e.g., gallic, vanillic, caffeic and coumaric acids, tyrosol, and hydroxytyrosol), complex compounds (e.g., secoiridoids, such as oleuropein and ligstroside), and lignans (e.g., pinoresinol and 1-acetoxypinoresinol). Total polyphenol content is highest in EVOO as opposed to concentrations in the other subtypes of olive oil [117,118]. EVOO has been associated with beneficial effects on human health, including the prevention of CV diseases, which is attributable to its composition that gives it antioxidant and anti-inflammatory properties [119]. Virgin olive oil also arises from the first pressing of olives, albeit with acidity levels below 2%. Virgin olive oil has also hydrophilic phenols, including phenolic acids and alcohols, flavonoids, lignans, and secoiridoids in lower concentrations as compared to EVOO and is apparently not as beneficial upon health as that of EVOO [117]. Even so, perhaps because it is more easily available, virgin olive oil is highly recommended in the literature [120]. The refinement of olive oil is produced using acids, alkalis, and heat to extract as much oil as possible from the olive pulp that remains after the first pressing. This processing renders refined oil higher in fat and acidic content than EVOO and virgin olive oil. Therefore, refined olive oil is deprived of optimal taste, aroma, and natural antioxidants and anti-inflammatory compounds [116]. Pomace oil is a by-product of EVOO. Olive skins, pulp, and seeds are heated, and the oil that remains is extracted, using hexane as a solvent. Pomace oil has few bioactive compounds and hence scarce antioxidant actions. Other types of oil produced through poor practices, such as lampante oil, are not recommended for human use, unless they are refined. Olive oil is composed mainly of triacylglycerols and contains small quantities of free fatty acids, glycerol, pigments, phosphatides, sterols, and flavor compounds. It has a high proportion of unsaturated and a low proportion of saturated fats. Olive oil consists of about 15% saturated fats including palmitic acid and stearic acid and about 85% unsaturated fats (70% oleic acid, a monounsaturated omega-9 fatty acid, 15% linoleic acid, an omega-6 PUFA, and 3.5% palmitoleic acid). In fact, olive oil contains more oleic acid and less linolenic acids (i.e., more MUFA than PUFA) than other vegetable oils; it is free of trans fatty acids. Several epidemiological observational studies have indicated that a higher proportion of MUFA in the diet may be linked with a reduction in the risk of CV disease [121,122]. The flavonoid polyphenols in olive oil are natural antioxidants that contribute to a bitter taste, astringency, and resistance to oxidation. The major phenolic compounds identified and quantified in olive oil—that is, simple phenols (e.g., hydroxytyrosol, tyrosol), secoiridoids (e.g., oleuropein, aglycone of ligstroside, and their respective decarboxylated dialdehyde derivatives), and lignans—have notable antioxidant properties. A high consumption of EVOO, particularly rich in these compounds, is associated with a diminished risk in colon, breast, and skin cancer in general and, due to the antioxidative effects of those components, there is a beneficial effect on coronary heart disease incidence as well as healthy aging [123]. Nevertheless, the polyphenol content is determined by many factors (e.g., variety of olives used, environmental factors, time of harvest, and extraction and storage conditions) [124].

Regarding the consumption of EVOO, a study including 6947 participants in the Three Cities Study cohort reported that an intensive use of EVOO slowed cognitive decline during the four years of follow-up. Compared with those participants who never used EVOO, participants with moderate or intensive EVOO use showed an improvement in visual memory and verbal fluency [125]. In the same cohort, analysis of 1329 older participants with high consumption of EVOO and other plant-derived polyphenols, including stilbenes, flavonoids, and lignans, among others, found that the risk of dementia was reduced by 50% in multivariable-adjusted models [126]. Vals-Predet et al. in a sample of 334 older participants of the PREDIMED trial at high risk of CV disease reported that after four years of a MedDiet supplemented with EVOO, memory scores, frontal cognition, and global cognition scores (including MMSE, Rey Auditory Verbal Learning Test, Animals Semantic Fluency, Digit Span subtest from the Wechsler Adult Intelligence Scale, Verbal Paired Associates from the Wechsler Memory Scale, and the Color Trail Test) declined less compared to the control diet, in whom the scores decreased significantly from baseline values [29]. In the PREDIMED-Navarra trial involving 522 participants at high CV risk, those receiving supplemented EVOO or mixed nuts showed higher cognitive scores and improved cognition vs. those in the control group. Participants in the group supplemented with EVOO had fewer cases of MCI compared to control participants following a low-fat diet [30,127].

Studying possible mechanisms that can explain the effects of EVOO, animal models of AD (senile SAMP8) fed with EVOO had improved learning and memory (T-maze foot shock avoidance and one-trial novel object recognition) associated with better profiles of circulating and brain oxidative stress markers [128]. Likewise, aged rats fed with EVOO showed improved brain biochemical parameters, memory, and motor coordination to levels similar to those of young animals by controlling oxidative stress, by activation of glutathione reductase, and by enhancing superoxide dismutase [129,130].

In summary, EVOO seems to have numerous health benefits shown in experimental and clinical trials. EVOO consumption seems to be neuroprotective as a crucial component of the MedDiet. However, there is not yet strong evidence for the effects of EVOO in isolation on cognitive decline and dementia, because there are very few clinical studies. However, EVOO should be tested in RCTs for the prevention of cognitive deterioration.

### 3.3. Nuts

Tree nuts provide macronutrients, micronutrients, and phytochemicals that may affect several pathways in AD pathogenesis such as amyloidogenesis, tau phosphorylation, oxidative stress, cholinergic pathways, and other non-target mechanisms including cholesterol lowering and anti-inflammatory properties, as well as effects on neurogenesis [131,132]. Among nuts, walnuts contain the largest amount of free and total polyphenols, which are followed by Brazil nuts and almonds [131]. Phytochemicals contained in tree nuts comprise carotenoids, phenolic acids, phytosterols and polyphenolic compounds such as flavonoids, proanthocyanidins, and stilbenes, as well as phytates, sphingolipids, alkylphenols, and lignans [132]. The phytochemical content of tree nuts can vary considerably by nut type, genotype, pre- and post-harvest conditions, as well as storage conditions. Although the beneficial effects of nuts on cardiometabolic diseases have been well established [133,134], less is known about the effects of nuts on cognitive well-being. Many of the biological pathways are common; hence, it is plausible that diets rich in nuts might be beneficial in ameliorating other age-related conditions as well.

Most studies on the effects of tree nuts on cognition or AD have been performed in preclinical studies in experimental animal models or in cell cultures [135]. While tree nut phytochemicals are bioaccessible and bioavailable in humans [132], the number of intervention trials conducted in humans to date is limited, while most of the evidence in humans comes from observational studies.

Considering the available human studies together—those designed specifically for nuts and those considering them within a dietary pattern—there are nineteen studies conducted in countries from different world regions (Europe, USA, Asia), of which there are thirteen observational and six interventional studies, reporting nut consumption effects and cognitive function in older adults. From the seven observational studies (three cross-sectional and four prospective) [136,137,138,139,140,141,142] specifically investigating nuts consumption and cognitive function, only one study [137] did not find significant differences in the cognitive test scores for participants with high vs. low nuts consumption after adjusting for confounders. The remaining six studies reported a significant positive association of various cognitive test and nuts consumption. The other six studies (three cross-sectional and three prospective) [56,143,144,145,146,147] investigated the associations of dietary patterns including nuts and cognitive function of older adults. It is worth clarifying that in these studies, it is not possible to attribute the findings to the intake of nuts specifically. Nevertheless, only one long-term study (nine-year follow-up) including 6000 older women did not find significant associations between nut consumption and global cognition or verbal memory [146]. The remaining five studies reported a lower risk of incident cognitive impairment or better overall cognition and verbal memory with a higher consumption of nuts.

As regards intervention studies investigating the effects of nut supplementation on the cognitive functioning of older adults, there are three studies available [148,149,150]. A former pilot study involving participants with MCI with a diet supplemented with one Brazil nut daily (about 5 g/day containing an estimated 288.75 µg/day of selenium) for six months found improvements in verbal fluency and constructional praxis scores but no difference in global cognition (two of six subsets of neuropsychological battery tests were improved) [148]. Two more recent and larger studies supplementing diets with almonds [149] or walnuts [150] as 15% of total daily energy intake did not observe any significant differences in cognitive performance or mood after the intervention. Three more studies including nuts as part of a MedDiet intervention [29,30,151] reported no benefit in a battery of cognitive tests [151], significant improvements in MMSE scores and clock drawing test [30], and improvements in a composite memory score but not in frontal and global cognitive function [29], all after adjustment for confounders. As it happens with other dietary components, in the interventional studies that used an overall dietary pattern approach, nuts consumption was only one component of the overall interventions, making it difficult to assess their independent effects.

In summary, most observational studies reported positive associations between nut consumption and cognitive function in older adults, while almost all interventional studies failed to demonstrate the benefits of nut supplementation (alone or as part of an overall dietary pattern) on cognitive function measurements. In general, observational studies were larger and of longer duration compared to intervention studies. It is possible that the benefits of nuts consumption on cognition may require very long-term habitual nut consumption. Nevertheless, the intervention studies are still few. Hence, larger RCTs that last longer are warranted for definitive conclusions.

### 3.4. Berries

Berries generally are rich in flavonoids including the flavan-3-ols catechin and epicatechin, the flavanols kaempferol and myricetin, and the anthocyanins delphinidin and petunidin [131]. In studies conducted in experimental animals and cellular cultures, it has been shown that flavonoids are neuroprotective and can slow brain aging and cognitive decline through a number of potential mechanisms, including the suppression of microglia-mediated inflammation as well as blood pressure and oxidative stress decrease with a consequent reduction of vascular risk, which is facilitated in part through the production of neuronal and inducible nitric oxide [152,153,154].

Specific studies assessing the possible cognitive effects of berries have been conducted experimentally, supporting the role of berry-rich diets in motor function, working memory, and increased neurogenesis [155].

Analyses of data from 16,010 participants from the observational Nurses’ Health Study aged ≥70 years found that a greater consumption of blueberries and strawberries in the long-term were significantly associated with slower rates of cognitive decline comparing extreme categories of consumption after adjusting for multiple potential confounders [156]. There are some small clinical studies on the effects of the consumption of berries or berry juices on cognitive parameters, which are detailed below.

A systematic review aiming to identify studies on food-based anthocyanin consumption (i.e., berry juices) and cognitive outcomes in human intervention trials included seven studies, comprising four acute trials and three longer-term interventions (twelve to sixteen weeks). Six of seven studies reported improvements in either, a single or multiple cognitive outcomes, including verbal learning and memory, after anthocyanin-rich food consumption. However, the authors found important methodological limitations due to the diversity of the studies, most of them very small trials, that prevented the pooling of data for quantitative analysis. Therefore, they concluded that even if food-based anthocyanin consumption seems promising, adequately powered studies are still needed [157]. Another systematic review evaluated RCTs investigating the effects of blueberries and blueberry products on cognition. Eleven articles (that included twelve studies) were identified; nine studies used freeze-dried blueberries, two studies used whole blueberries, and one study used blueberry concentrate. Eight studies reported an improvement in cognitive performance, particularly short- and long-term memory and spatial memory, after blueberry consumption or supplementation at various doses and time lengths. However, considerable differences in the study design, dosages, and anthocyanin content hinder between-study comparison [158]. A double blinded, chronic intervention RCT investigated the effect of two blueberry formulations (whole wild blueberry powder at 500 mg (WBP500) and 1000 mg (WBP1000) and a purified extract at 100 mg (WBE111)) for six months vs. placebo on cognitive performance in 120 older adults (aged 65 to 80 years). The study results indicated that a three-month intervention with WBE111 facilitated better episodic memory performance (i.e., delayed word recognition on the Reys Auditory Verbal Learning Task) and reduced CV risk factors over 6 months [159]. Another small RCT involving thirteen men and twenty-four women, aged 60 to 75 years, randomized participants to consume either freeze-dried blueberry (24 g/d, equivalent to 1 cup of fresh blueberries) or a blueberry placebo for ninety days. Participants in the blueberry group showed significantly fewer repetition errors in the California Verbal Learning test and reduced switch cost on a task-switching test across study visits compared to controls. However, no improvement in gait or balance was observed [160]. Another small trial included healthy older adults (*n* = 12 intervention; *n* = 14 control) randomized to consume either 30 mL of blueberry concentrate providing 387 mg anthocyanidins or isoenergetic placebo for twelve weeks undertaking a battery of cognitive function tests and fMRI pre- and post-supplementation. Significant increases in brain activity and in working memory were observed in response to blueberry supplementation relative to the placebo group in brain areas associated with cognitive function [161]. In another study, 40 healthy 50 to 70-year-old participants were provided a berry beverage based on a mixture of berries (150 g blueberries, 50 g blackcurrant, 50 g elderberry, 50 g lingonberries, 50 g strawberry, and 100 g tomatoes) or a control water-based beverage (matched with respect to monosaccharides, pH, and volume) daily during five weeks in a randomized crossover design. Cognitive tests included tests of working memory capacity, selective attention, and psychomotor reaction time, while cardiometabolic parameters included blood pressure, fasting blood glucose, insulin, blood lipids, inflammatory markers, and markers of oxidative stress. The berry intervention significantly reduced total- and low-density lipoprotein (LDL) cholesterol vs. baseline and vs. the control beverage. The control beverage increased blood glucose and tended to increase insulin concentrations vs. baseline, and it also tended to increase insulin concentrations vs. the berry beverage. Participants performed better in the working memory test after the berry beverage when compared to after the control beverage. No changes in the cognitive tests were observed [162]. Krokorian et al. conducted another small study in a sample of nine older adults with early memory changes randomized to daily consumption of wild blueberry juice. After twelve weeks of intervention, the authors observed improved paired associate learning and word list recall vs. baseline values. They also compared the memory performances of participants receiving the intervention with a demographically matched sample who consumed a berry placebo beverage and observed comparable results for paired associate learning. The authors concluded that this preliminary study suggests that moderate-term blueberry supplementation can confer neurocognitive benefit, but larger comprehensive human trials are warranted [163]. In a 24-week double-blind RCT, older men and women received daily fish oil or blueberry or both and neuropsychological assessment (Dysexecutive Questionnaire, Trail-Making Test, Controlled Oral Word Production, and alternate forms of the Hopkins Verbal Learning Test to assess new learning and long-term memory with scores representing cumulative learning, delayed recall, percentage recalled, and item discrimination in recognition memory). Total urinary anthocyanins did not differ between the groups after supplementation. Both fish oil and blueberry groups reported fewer cognitive symptoms, and the blueberry group showed improved memory discrimination. However, combined fish oil plus blueberry treatment was not associated with cognitive enhancement [164]. A double-blind RCT was conducted among 215 healthy older adults (aged 60 to 70 years) receiving 600 mg/d of a polyphenol-rich extract from grape and blueberry (PEGB) (containing 258 mg of flavonoids) or a placebo for six months. Cognitive tests included the Cambridge Neuropsychological Test Automated Battery Paired Associate Learning (CANTAB PAL), a visuospatial learning and episodic memory test, as the primary outcome, while secondary outcomes included verbal episodic and recognition memory (VRM) and spatial span (SSP) working memory. There was no significant effect of PEGB on the PAL on the whole cohort, but a subgroup with advanced cognitive decline responded positively to the PEGB. VRM-free recall was improved after PEGB supplementation. Urinary concentrations of specific flavan-3-ols metabolites were associated, at the end of the intervention, with the memory improvements [165].

In summary, available data seem to indicate possible neuroprotective effects of berries or their products. However, the available results come from small studies, and there are still no strong data from large RCTs of duration long enough to draw definitive conclusions or recommend the consumption of berries to improve cognitive functions.

### 3.5. Coffee

There is evidence from in vitro studies indicating that caffeine has antioxidant properties [166] and from experiments in AD animal models showing amyloid-beta suppression [167]. The decrease in amyloid-plaques was associated with greater levels of phosphor-cyclic amp-response element binding protein (CREB), protein kinase A activity stimulation, and a reduction in the expression of phosphor-c-Jun N-terminal kinase (JNK) and phosphor-extracellular receptor kinase (ERK) in mouse models of AD, which may activate brain survival cascades [167,168]. In the short-term, coffee and caffeine are recognized stimulants to memory and cognition. Yet, the evidence on long-term effects is scarce. A case-control study reported that elevated serum levels of caffeine were associated with lack of progression to dementia in a sample of 124 older adults with MCI [169]. In the Cardiovascular Risk Factors, Aging and Dementia (CAIDE) study, coffee drinkers had lower risk of developing AD and dementia; the lowest risk (65% reduction) was reported for participants consuming three to five cups of coffee daily after a 20-year follow-up [170]. Analysis of data from the Honolulu-Asia Study reported that the highest caffeine intake was associated with a lower risk of post-mortem neuropathological dementia lesions vs. the lowest caffeine intake. Nevertheless, coffee and caffeine intake during midlife were not associated with AD, vascular dementia, cognitive decline, or neuropathological lesions [171]. A study conducted in Portugal reported an association between caffeine intake with slower cognitive decline [172], while another study conducted in France did not report any association [173]. A longitudinal study by Arab et al. showed a lower risk of cognitive decline among participants with higher coffee intake, but there was no dose response [174]. A Finnish study showed no relationship [175]. A meta-analysis of studies evaluating the association of caffeine intake and cognitive deterioration did not find significant effects [176]; the studies included were highly heterogeneous.

Recent investigations continue to provide inconsistent results. A meta-analysis aiming to investigate the dose–response relationship between alcohol, coffee, or tea consumption and cognitive deficits including prospective cohort studies or nested case-control studies (*n* = 29) from America, Japan, China, and some European countries published up to June 2020 found that a low consumption of coffee reduced the risk of any cognitive deficit (<2.8 cups/day) or dementia (<2.3 cups/day) [177]. Conversely, a study exploring the effect of lifetime coffee consumption on the volume of white matter hyperintensities (VWMH) in late life examining 492 cognitively normal community-dwelling men and women (mean age 73.4 ± 6.7 years) from the Korean Longitudinal Study on Cognitive Aging and Dementia found that higher cumulative lifetime coffee consumption was associated with significantly higher log VWMH in both sexes. Participants consuming >2 cups of coffee per day on average in their lifetime showed higher log VWMH in late life than those who consumed less. These findings suggest that prolonged high coffee consumption may be associated with the risk of WMH in late life [178]. Likewise, a prospective cohort study from Japan examining the association of average green tea and coffee consumption in the previous year with repeated MMSE scores in 620 men and 685 women from The National Institute for Longevity Sciences, Longitudinal Study of Aging (NILS-LSA) did not observe any association between coffee consumption and incident cognitive decline (MMSE < 27) after a mean follow-up of 5.3 years, while it was significant for tea consumption [179].

There are also some positive but contrasting results, such as those reported by a study investigating the entity of brain AD pathologies (i.e., cerebral beta-amyloid deposition and WMH). Among 411 non-demented older adults, lifetime coffee intake of ≥2 cups/day was significantly associated with a lower beta-amyloid positivity vs. coffee intake <2 cups/day, after adjusting for confounders. However, lifetime or current coffee intake was not related to hypometabolism, atrophy of AD-signature region, and WMH volume [180]. Since coffee is rich in polyphenols (i.e., caffeine, diterpenes, melanoidins, and trigonelline), which can stimulate brain activity, a cross-sectional analysis of data from participants of the PREDIMED-plus study who completed the MMSE (*n* = 6427; mean age = 65 ± 5 years; with metabolic syndrome) or a battery of neuropsychological tests explored the association of coffee consumption and total dietary caffeine intake with the risk of poor cognitive functioning. Total coffee consumers and caffeinated coffee consumers had better cognitive functioning than non-consumers after adjusting for potential confounders (OR 0.63; 95% CI 0.44–0.90 and OR 0.56; 95% CI 0.38–0.83, respectively). These associations were not observed for decaffeinated coffee consumption. Participants in the highest tertile of total dietary caffeine intake had significantly lower odds of poor cognitive functioning than those in the lowest tertile of caffeine intake [181]. There is no specific evidence on the effects of coffee on cognitive decline in RCTs.

In conclusion, there is no consistency in the literature on the association of positive effects of coffee on long-term cognition. More research is still needed on this important subject.

### 3.6. Cocoa

Cocoa and its derived products are a rich source of flavonoids, which as discussed earlier have shown CV and cognitive benefits [182]. Small short-term and chronic trials have suggested some neuroprotective properties. A study involving eighteen persons aged 50–65 years showed significant increases of brain perfusion in the anterior cingulate cortex and the central opercular cortex of the parietal lobe in participants receiving a high flavanol (494 mg) cocoa drink, which was measured with fMRI [183]. Another small study involving 34 cognitively normal persons with an average age of 72 ± 6 years reported a significantly increased blood flow velocity in the middle cerebral artery after one (8%) and two weeks (10%) of flavanol-rich cocoa consumption, which was measured with transcranial ultrasound [184]. An RCT of 41 healthy persons (age range 50–69 years) who consumed a diet containing high quantities of cocoa-derived flavanol for 3 months reported enhanced dentate gyrus function, as measured by cognitive tests and fMRI [185]. A longitudinal study among 531 over-65 participants followed for a median of 48 months reported that chocolate intake was associated with a 41% lower risk of cognitive decline in multivariate-adjusted analysis [186]. A double-blind RCT was conducted aiming to explore the effect of cocoa flavanol drink consumption (993 mg/d, 520 mg/d, or 48 mg/d for 8 weeks) on cognitive performance in 90 cognitively intact older adults. Cognitive function was assessed at baseline and after 8 weeks by MMSE, Trail-Making Test (TMT) A and B, and the Verbal Fluency Test (VFT). MMSE score response was similar for the three doses, while TMT A and B and VFT improved after consumption of the high and intermediate doses vs. the lowest dose of cocoa flavonol drink. In addition, the authors reported significant improvements in insulin resistance, blood pressure, and lipid peroxidation for the high and intermediate dose vs. the lowest dose [187]. Conversely, another double-blind RCT reported less significant results. This RCT involved 40 young participants (mean age = 24.1 ± 4.5) to evaluate the effects of acute (same-day) and sub-chronic (daily for four weeks) 250 mg cocoa supplementation on mood and mental fatigue, cognitive performance, and CV functioning (peripheral and central blood pressure and cerebral blood flow). Cognition was assessed with repeated 10-minute cycles of the Cognitive Demand Battery (CDB), a Rapid Visual Information Processing task, a mental fatigue scale over the course of half an hour, and the Swinburne University Computerized Cognitive Assessment Battery (SUCCAB). Consumption of cocoa significantly improved only self-reported mental fatigue and performance on the Serial Sevens task in cycle one of the CDB in the acute time point. No other significant effects were found [188].

The dentate gyrus (DG), a region in the hippocampal formation whose function declines in association with human aging, is considered a possible source of age-related memory decline. An RCT using a high-resolution variant of fMRI to map the precise site of age-related DG dysfunction studied 37 healthy adults (aged 50–69 years) who consumed either a high (900 mg/d) or a low (45 mg/d) cocoa flavanol-containing diet for 3 months. A high-flavanol intervention was found to enhance DG function, as measured by fMRI and by cognitive testing, establishing that DG dysfunction is a driver of age-related cognitive decline and suggesting non-pharmacological means for its amelioration [185].

A review published in 2017 on available human studies specifically aiming at evaluating the effects of acute and chronic administration of cocoa flavanols on different cognitive domains, although still at a preliminary stage, reported a tendency to dose-dependent improvements in general cognition, attention, processing speed, and working memory. However, the number of studies on cognitive findings is still limited, reporting mixed results in contrast to studies on physiological responses to flavonoid supplementation such as vasodilation, both at peripheral and central levels, which have been consistently replicated. Discrepancies are thought to be the result of methodological differences (i.e., dose, form, and timeframe of the cocoa flavanols administration, as well as in the length and cognitive load of the experimental tasks). Nevertheless, the authors state that the findings point to cocoa as a new interesting nutraceutical tool to potentially protect human cognition and counteract different types of cognitive decline, thus encouraging further investigations [189].

The mechanisms proposed to explain the potential benefit on cognition of cocoa and cocoa products are multiple, ranging from its antioxidants actions [190,191,192] to direct interactions with cellular signaling that promote neurogenesis, neuronal function, and brain connectivity [193,194,195] as well as blood-flow improvement and angiogenesis in the brain and sensory systems [196,197,198]. Other active components of cocoa, such as methylxanthines (caffeine and theobromine), may be considered. Caffeine, as mentioned above, has been extensively studied, while theobromine has been examined less. Animal and human studies have suggested a potential neuroprotective action of long-term consumption of theobromine through a reduction of beta amyloid pathology. The potential action of theobromine alone and associated with caffeine or other cocoa constituents on cognitive modulation is underexplored to date, and future studies are needed to shed light on this promising molecule [199].

In summary, the preliminary evidence shown above, even if not definitive and still inconsistent, underscores the need for further well-design and large RCT on cocoa and cocoa-derived products to better elucidate their neuromodulatory properties and potential cognitive effects.

### 3.7. Garlic

In experimental animals, extracts of garlic have exhibited antioxidant properties and protective actions against amyloid-beta-induced neurotoxic effects [200]. In vitro studies have reported that allicin, an organosulfur compound contained in garlic, inhibited cholinesterase enzymes and upregulated brain acetylcholine concentrations [201]. In experimental animals, S-allyl cysteine, the active compound of aged garlic extract, has shown a mitigation of LPS-induced cognitive deficits via the attenuation of oxidative stress, neuroinflammation, astrogliosis, and acetylcholinesterase activity [202]. However, no clinical trials for these compounds are available. There are some negative reports, such as those from the Doetinchem Cohort Study comprising 2613 participants aged 43–70 years, in which a higher consumption of allium (onion, garlic, and leek) was associated with worse scores on speed of cognitive processes and cognitive flexibility in cross-sectional analyses, and there was no association with cognitive decline in longitudinal analyses [142]. More recent experimental studies confirm that garlic extract may be effective in alleviating cognitive impairment and neuropathology in AD animal models [203,204], but there is no evidence of possible effects on cognition in human studies.

### 3.8. Tea

In Asian cultures where tea is largely consumed, it is traditionally considered a cognitive enhancer. Acute effects of tea consumption on mood and cognitive performance have been reported in some studies and have been linked to antioxidants contained in tea, such as epigallocatechin-3-gallate (EGCG), L-theanine, and caffeine [205]. In addition, it has been suggested that the neuroprotective actions of tea consumption may be mediated by inhibition of acetylcholinesterase and regulation of stress hormones [206]. Nevertheless, there is no definitive evidence on tea consumption neuroprotective actions due to inconsistent results of available studies [207]. Tea bioactive components, i.e., L-theanine and EGCG, may have shown anti-amyloidogenic and antioxidant properties in vitro, but the evidence for their use in humans as nutraceuticals is limited; hence, their use in the clinical practice is not currently recommended.

Recently, a prospective cohort study analyzing data from 1305 cognitively competent participants at baseline of the NILS-LSA in Japan, aged 60–85 years, examined the mean consumption of green tea and coffee in the previous year in relation to cognitive decline assessed with repeated measurements of MMSE score (up to six times biennially). During follow-up (mean of 5.3 ± 2.9 years), 432 participants had incident cognitive decline (MMSE < 27); in multivariable-adjusted Cox proportional hazard regression, participants who consumed green tea once/day, 2–3 times/day, and ≥4 times/day had a progressively significant lower risk of incident cognitive decline compared to participants who consumed green tea < once/day. No significant association was found between coffee intake and cognitive decline [179].

Since the results of studies in humans on alcohol, coffee, and tea consumption in relation to cognitive decline have been incongruous, a recent meta-analysis of 29 prospective cohort studies or nested case-control studies in a cohort from different countries worldwide aimed to find the dose–response relationship between alcohol, coffee, or tea consumption and cognitive deficits. The dose–response relationships showed that compared to non-drinkers, green tea consumption was a significant protective factor for cognitive health; one cup of tea per day was associated with a 6% reduction in the risk of cognitive deficits [177]. Nevertheless, the available studies are all observational, and there is no solid evidence of protective effects of tea consumption on cognitive decline.

### 3.9. Alcohol

We have mentioned how both risk and protective factors for CV disease are also risk/protective factors for cognitive impairment and dementia. The MedDiet dietary pattern includes the possibility of consuming light to moderate amounts of alcohol (5–25 g/day for women and 10–50 g/day for men), which has been found to be associated with a lower risk of total mortality, type 2 diabetes, coronary heart disease, stroke, and heart failure [208]. The evidence of wine’s cardioprotective effects emerged in earlier studies [209]. However, the proposed CV protective actions of alcohol have been lately a matter of debate with evidence showing an inverse association of alcohol consumption with coronary heart disease but also with an increased risk of different types of stroke [210], which is very relevant for the development of cognitive decline and dementia. It is clear that heavy drinking (over 4 drinks/day) represents a risk factor for CV and other chronic diseases [211] and is a leading cause of premature deaths in the USA [212]. Supporting the message that there is no safe level of alcohol consumption, studies based on Mendelian randomization analyses in mega-cohorts have questioned the CV benefits of alcohol consumption [213]. However, the effects of alcohol consumption on the risk of cognitive deterioration appear to be strongly modified by the presence of APOE ε4 allele. Analyses of data from the Epidemiology of Vascular Aging (EVA) prospective study involving 1,389 participants aged 59-71 years followed for 4 years and conducted in France found that alcohol drinking was associated with a decreased risk of cognitive deterioration in non-APOE ε4 carriers, whereas an opposite association was observed in APOE ε4 carriers [214]. Other data have accumulated confirming that moderate alcohol consumption may be beneficial for cognition. A recent meta-analysis of observational studies concluded that light to moderate alcohol consumption is associated with a reduced risk of dementia, whereas both abstinence and heavy drinking are associated with a higher risk of dementia [215]. Similar results were obtained in the Whitehall II cohort study follow-up [216]. Furthermore, it is well known that both abstinence and excess alcohol are associated with increased risk of CV disease and diabetes, which are established links with dementia. Concerning cognition, there is also abundant epidemiological evidence that low to moderate alcohol consumption is associated with better cognitive function than abstinence or excess drinking [217,218,219,220].

For red wine, it has been suggested that its composition comprising not only alcohol but also bioactive compounds (polyphenols) can impact oxidative stress and chronic inflammation [221]. Nevertheless, research on resveratrol, the most intensely studied wine polyphenol, has not demonstrated that the neuroprotective effects obtained in experimental animals are replicable in humans, as discussed below (Section 3.14).

A meta-analysis of twenty-nine prospective studies aiming to explore possible dose–response relationship between alcohol, coffee, and tea consumption and cognitive deficits found that compared to non-drinkers, low consumption (<11 g/d) of alcohol could reduce the risk of cognitive deficits or dementia, but there was no significant effect of heavier drinking (>11 g/d) [177]. However, another recent review aiming to critically summarize the main relevant studies to clarify the relationship between wine drinking and AD, as well as how frequency and/or amount of drinking may influence this relationship, concluded that no definitive results can clarify if light to moderate alcohol drinking is detrimental to cognition and dementia, or if alcohol intake could reduce the risk of developing AD [222]. A study on data from the Baltimore Longitudinal Study of Aging including ten cognitive scores, spanning various domains of cognition, found mixed effects of alcohol on domains of letter fluency, attention, and working memory with heterogeneous results depending on analyses by sex and baseline age, concluding that further longitudinal large studies are still needed to allow certain conclusions [223]. A recent dose–response meta-analysis including six prospective studies (*n* = 4244) with data on at least three levels of alcohol exposure found that heavy alcohol intake (>14 drinks/week) was significantly associated with higher risk of progression to dementia in people with MCI, while there was a nonlinear relation between alcohol intake and risk of this progression. Drinking over 16 drinks/week would significantly further elevate the progression to dementia risk [224].

In summary, there are still contrasting results regarding the protective effects of low/moderate alcohol consumption on the risk of cognitive impairment and dementia. Certainly, there is accumulated evidence of benefit, but there are also other studies that fail to reach definitive conclusions. The point on which there is general agreement is that excessive alcohol consumption can be detrimental for cognitive functions, and besides, in some cases, even moderate consumption can engender increased risk of drowning, violence, and injuries from car accidents and falls, and with a higher risk of breast cancer [225].

### 3.10. Curcumin

The rhizome of *Curcuma longa*, part of the curry spice widely used traditionally in Asian cuisine, contains the polyphenolic compound turmeric curcumin, which is used as therapy for various conditions in traditional Indian medicine and a potent antioxidant [226]. It has been reported that older healthy people who consumed curry frequently had better cognitive performance vs. non-consumers of curry [227], while the Indian population that widely used curry seem to have a lower prevalence of AD compared to the US population [228].

A number of experimental studies have shown the potent anti-oxidant and anti-inflammatory properties of curcumin and its protective effects against AD in animal models [229], while few RCTs and case reports are available [230,231,232,233,234]. A study involving thirty-four AD patients receiving either 1 or 4 g/d of curcumin or placebo for six months found no significant effects in MMSE [230]. Another RCT included thirty-six patients with dementia receiving 2 or 4 g/d of curcumin C3 complex (95% of curcuminoids) or placebo for twenty-four weeks, which was followed by a 48-week open-label trial with curcumin C3 complex for the placebo arm. There were no significant differences between treatment groups and placebo in any of the cognitive scores used (ADAS-Cog, Neuropsychiatric Inventory –NPI–, ADCS-ADL scale, MMSE) or cerebrospinal fluid markers [231]. A case report article described three patients with dementia and severe behavioral and psychological symptoms who had a remarkable improvement of these symptoms (irritability, agitation, anxiety, and apathy) after treatment with 100 mg/day of curcumin and donepezil for twelve weeks with significantly decreased NPI scoring [232]. A double-blind RCT examined the acute (1 and 3 h after a single dose), chronic (4 weeks), and acute-on-chronic (1 and 3 h after single dose following chronic treatment) effects of solid lipid curcumin formulation (400 mg as Longvida^®^) on cognitive function, mood, and blood biomarkers in sixty healthy adults (age range: 60–85 years). Curcumin significantly improved performance on sustained attention and working memory tasks (digit vigilance task accuracy and serial three subtraction task) after one hour of administration compared with placebo. Following chronic treatment, working memory and mood (general fatigue and change in state calmness, contentedness, and fatigue induced by psychological stress) were significantly better vs. placebo. A significant acute-on-chronic treatment effect on alertness and contentedness was also reported [233]. Finally, another double-blind RCT investigated the ability of a curcumin formulation to prevent cognitive decline in a population of community-dwelling older adults. Ninety-six participants received either placebo or 1500 mg/d Biocurcumax^®^ for 12 months. A battery of clinical and cognitive measures was administered at baseline and after 6 and 12 months of treatment. The repeated measures analysis revealed an interaction for the Montreal Cognitive Assessment, which was driven by a decline in function of the placebo group at 6 months that was not observed in the curcumin treatment group. No differences were observed between the groups for all other clinical and cognitive measures. Thus, further longitudinal studies are required to investigate eventual modifications in cognitive outcome measures with curcumin administration, which may be improved if performed in conjunction with biological markers of neurodegeneration [234].

A recent review of preclinical and clinical studies evaluated the efficacy of curcumin in ameliorating and preventing age-associated cognitive decline also addressing the translational progress of preclinical to clinical efficacy. Results from preclinical studies consistently demonstrated that curcumin and its analogues were efficacious for various aspects of cognitive impairment and processes that contribute to age-associated cognitive decline. Results of the few published clinical studies were not univocal, but some continue to show promising results for curcumin’s use against cognitive decline but overall remained currently inconclusive. Both in vitro and in vivo studies have reported that curcumin can significantly decrease oxidative stress and systemic inflammation as well as hinder pathways that activate transcription factors associated with the enhancement of these processes. Thus, still further clinical studies are needed, perhaps including the evaluation of peripheral and cerebrospinal fluid biomarkers of dementia (e.g., amyloid and tau status) and behavioral markers of cognitive decline as well as targeting appropriate populations [235].

Turmeric supplements generally have low bioavailability, which is why new formulations have been proposed, such as polymeric micelles, polymer nanoparticles, nanogels, dendrimers, nanoemulsions, inclusion complexes, phytosomes, solid–lipid nanoparticles, curcumin nanoparticles, liposomes nanoparticles, liposomes, micelles, nanogel, dendrimers, nanoemulsions, inclusion complexes, and phytosomes with potential to reduce intestinal degradation and increase curcumin bioavailability, ultimately enhancing its efficacy throughout the body and the brain [229].

### 3.11. Omega-3 Fatty Acids

The PUFAs are crucial components of the neuronal cell membranes, which preserve membrane fluidity for synaptic vesicle fusion and neurotransmitter communication. PUFAs may be lipid messengers and precursors for signaling processes to promote protection or prevent neuronal damage [236]. A deficit of PUFAs in the hippocampus, cortex, and cerebellum has been reported in the aged brain, which may be worse in AD [237]. The most extensively studied PUFAs regarding cognitive deterioration are omega-3 long chain (LC) PUFAs with conflicting results. A systematic review concluded that omega-3 long chain (LC)PUFAs play a relevant role in the reduction of cognitive decline [238], while other studies have shown negative results. For example, the “Supplementation with Folate, vitamin B6 and B12 and/or Omega-3 fatty acids” trial, involving 1748 participants with a history of CV disease, did not find any significant effect of vitamin B and omega-3 PUFA supplementation on cognitive function [239]. A double-blind RCT including 302 cognitively intact persons older than 65 years receiving 1.8 g/d of eicosapentaenoic acid (EPA)-docosahexaenoic acid (DHA), 0.4 g/d EPA-DHA, or placebo for 26 weeks found no significant effects [240]. A meta-analysis of three trials comprising data from 3536 participants aged over sixty years with preserved cognitive performance at baseline supplemented with omega-3 PUFAs reported no significant effects on cognitive function [241]. A study conducted in China found that participants older than 65 years who consumed ≥100 g/week of fish had a reduction of near 65% in the mean annual rate of global cognitive decline with no associations among participants aged 55–64 years [242]. Likewise, the Multidomain Alzheimer Preventive Trial (MAPT) including 1525 participants found that a multidomain intervention plus omega-3 PUFAs supplementation, either alone or in combination, had no significant effects on cognitive deterioration over 3 years [243].

More recent analyses of data from 1445 MAPT participants explored the effects of PUFAs and the mentioned multidomain intervention on levels of intrinsic capacity (IC), which is a construct proposed by the WHO including the Geriatric Depression Scale (psychological); Short Physical Performance Battery (mobility); Z-score combining four tests (cognitive function); and handgrip strength (vitality). After three years, the IC Z-score decreased significantly among all groups with no significant differences between groups, confirming no effect of the intervention [244]. A recent cross-sectional study conducted in Australia investigated the relationship between erythrocyte omega-3 LCPUFA, DHA and EPA levels and their corresponding dietary intakes with cognition and physical function in a cohort of 142 community-dwelling older adults (60–85 years) at risk of dementia. Higher dietary DHA and EPA were associated with better global cognitive function, better attention/psychomotor composite scores, mobility (four-square step test), and gait speed. No associations were found between erythrocyte omega-3 LCPUFA and cognitive or functional performance measures [245]. A recent narrative review examined the available evidence on the association between DHA/EPA (blood testing or dietary intake) and brain volume in non-demented older adults aged 45 years or over. The search identified twelve studies: eight cross-sectional observational studies, three longitudinal cohort studies, and one RCT published between 2007 and 2019. The largest amount of evidence indicated that the hippocampus was most frequently involved in this association, with a higher volume associated with higher omega-3 levels. Most studies reviewed provided mixed findings regarding the presence or absence of the association of interest, and the findings were observed to be brain region-dependent. Hence, the authors concluded that the current evidence is still insufficient to formulate recommendations for omega-3 PUFA intake to support brain health specifically [246]. Another recent trial [247] and a meta-analysis [248] reported negative results. The small RCT (*n* = 33) evaluated the effect of supplementation with omega-3 fatty PUFAs (2.3 g/d) on diverse biomarkers analyzed in the cerebrospinal fluid (CSF) of patients with AD at baseline and after six months of treatment. There were no significant differences between the groups concerning the level of the different biomarkers in the CSF at baseline with a small but significant increase in only two of the multiple biomarkers measured. This increase in biomarkers did not correlate with MMSE score [247]. A systematic review and meta-analysis of thirty-eight RCTs (comprising 49,757 participants) with longer duration than twenty-four weeks assessed the effects of increasing omega-3, omega-6, or total PUFA on incident neurocognitive illness and cognition. The meta-analysis suggested no or very little effect of omega-3 LCPUFA on incident neurocognitive illness, new cognitive impairment, or global cognition. Effects did not change with sensitivity analyses, different doses, durations, intervention types, or replacements. Therefore, the authors concluded that omega-3 LCPUFA supplements do not help older adults protect against cognitive decline [248]. There are also some sporadic positive results. For example, among 285 participants with stable coronary artery disease on statin treatment randomized to 3.36 g/d EPA and DHA or none for 30 months assessing cognitive function at baseline and after 12 and 30 months with neuropsychological testing, participants on EPA/DHA treatment had significantly better scores vs. controls for verbal fluency, language, recall memory, and visual–motor coordination [249]. A post doc analysis of the placebo-controlled trial Folic Acid and Carotid Intima-media Thickness (FACIT) investigated the interaction between baseline omega-3 PUFA statuses and folic acid treatment on cognitive decline in 791 older adults aged 50–70 years. Participants received 800 microg/d of folic acid or placebo for 3 years, and global cognitive functioning and domain-specific functioning (episodic memory, information processing speed, executive functioning) were assessed at baseline and after 3 years. The efficacy of folic acid treatment on cognitive functioning was dependent on the omega-3 PUFA status: participants with a lower omega-3 PUFA status at baseline benefited from folic acid treatment, while individuals with a higher omega-3 PUFA did not, which may help explain the inconsistency in outcomes of B-vitamin supplementation trials on cognition [250].

In summary, the findings of studies on the effects of omega-3 PUFAs on cognition are hitherto mixed. Therefore, at present, there is still insufficient evidence to recommend this type of supplementation to improve cognitive performance or prevent cognitive decline.

### 3.12. Magnesium

There is substantial evidence showing that a deficit of magnesium (Mg) leads to increased free radicals production in various tissues, increased oxidative tissue damage, greater superoxide anion production by inflammatory cells, reduced antioxidant enzyme expression and activity, lessened cellular and tissue antioxidant concentrations, and augmented oxygen peroxide production [251,252]. Mg is crucial for synaptic conduction, N-methyl-D-aspartate (NMDA) receptor response to excitatory amino acids [253], inhibition of calcium channels, calcium influx, and glutamate release, and for the stability and viscosity of cell membranes [251]. Vasospasm has been reported in conditions of Mg deficiency; contrariwise, high Mg levels induce tone relaxation in cerebral arteries [254]. The previous results have aroused interest in studying the role of Mg in cognitive decline and dementia in recent years. Earlier investigations had shown that AD patients exhibited low serum Mg levels [255] and decreased Mg brain tissue concentrations in autoptic studies [256]. We found reduced plasma concentrations of ionized free Mg in AD patients [257] that were associated with the severity of the cognitive dysfunction. A study reported a negative significant association of serum Mg concentrations with two scales of AD clinical severity (Global Deterioration Scale and Clinical Dementia Rating), supporting the likely protective role of Mg on cognitive performance [258].

More recent analyses of observational studies have confirmed the association of Mg deficit with an increased possibility of incident cognitive decline. A systematic review of studies investigating Mg status was performed comparing AD with healthy controls (HCs) or controls with medical illness (MCs). Thirteen studies were included (AD: 559; HCs: 381; and 126 MCs). Compared to HCs, patients with AD had significantly lower Mg in cerebrospinal fluid (two studies) and hair (two studies). No differences between AD and controls were evident for serum Mg. Comparisons with MCs were hindered by the scarcity of studies including these patients [259]. Mg plays a critical role in vitamin D biosynthesis and metabolism [260]. Deficiencies in both nutrients, which is frequent in old age, have been associated with poor cognition. A study aimed to evaluate the associations of Mg intake and serum 25-hydroxyvitamin D [25(OH)D] concentrations with cognition and the interaction between these nutrients based on data from the National Health and Nutrition Survey (NHANES) 2011–2014 including 2466 participants aged over sixty years. Cognitive impairment was defined as a Digit Symbol Substitution Test (DSST) score lower than the lowest quartile. Higher total Mg intake was independently associated with higher DSST scores, especially among women, non-Hispanic whites, physically active participants, and those with sufficient serum 25(OH)D, although the interactions were not significant. The odds of cognitive impairment were significantly reduced with increasing intake of total Mg and higher level of serum 25(OH)D [261]. A random sub-cohort (*n* = 2063) from the Reasons for Geographic and Racial Differences in Stroke (REGARDS) US cohort was investigated regarding baseline serum Mg concentration and incident cognitive impairment identified with the Six-Item Screener. After adjustment for potential confounders, an inverse threshold association between serum Mg level and incident cognitive impairment was observed. Compared to those with the worst level of hypomagnesemia (<0.75 mmol/L), the relative odds of incident cognitive impairment were reduced by 41% in the intermediate level of serum Mg (0.75–< 0.81); a higher serum Mg level did not provide further benefits. Thus, sufficient Mg status within the normal range may be beneficial to cognitive health in the US population [262]. Another study investigated the associations of plasma Mg concentration with AD and non-AD dementia. Plasma Mg concentrations were measured in 102,648 participants from the Copenhagen General Population Study. Multifactorially adjusted hazard ratios for non-AD dementia were 1.50 (95% confidence interval (CI):1.21–1.87) for the lowest and 1.34 (CI: 1.07–1.69) for the highest vs. the fourth quintile (reference) of plasma Mg concentrations. Diabetes, cumulated smoking, stroke, and systolic blood pressure mediated 10.4%, 6.8%, 1.3%, and 1.0%, respectively, in the lowest quintile, whereas stroke mediated 3.2% in the highest quintile. No associations were observed for AD. The lowest risk observed for non-AD dementia was at a concentration of 2.07 mg/dL. No association was observed for AD. Mediation analysis suggested that diabetes may be in the causal pathway between low plasma Mg concentrations and high risk of non-AD dementia, while cumulated smoking, stroke, and systolic blood pressure played minor mediating roles [263]. Data from 12,040 cognitively intact participants at baseline from the Atherosclerosis Risk in Communities (ARIC) observational study were analyzed to study the association of serum Mg with cognitive functioning capabilities assessed repeatedly. There were 2519 cases of incident dementia over a median follow-up period of 24.2 years. Participants in the lowest quintile of serum Mg had a 24% higher rate of incident dementia compared to those in the highest quintile of Mg. No relationship was found between serum Mg and cognitive decline in any cognitive domain. Hence, according to these results, low midlife serum Mg was associated with increased risk of incident dementia but did not appear to impact rates of cognitive decline [264].

In summary, at present, there is accumulated evidence supporting a role for Mg in cognitive decline, both as an antioxidant and neuroprotective agent as well as in association with the incidence of dementia in a number of observational studies. However, so far, there are no trials testing whether Mg supplementation can directly be preventive or therapeutic in cognitive disorders; thus, the protection of Mg supplementation for the development of AD remains to be further elucidated in well-designed and conducted RCTs.

### 3.13. Ginkgo biloba

The leaves of the tree *Ginkgo biloba* (Gb) native to China are a popular herbal medicine in Traditional Chinese Medicine, whose extracts have been used for various disorders including cognitive disorders, hypertension, coronary heart disease, and cerebral ischemia [265,266,267,268]. In animal models [267,269] and in vitro [270], the extract of Gb leaves has been reported to attenuate neuronal damage, in particular that induced by amyloid-beta [271,272,273,274] and even to have beneficial effects on cognition in combination with donepezil in animal models of AD [275,276]. The chemical constituents of Gb comprise flavonoids, terpene lactones, and ginkgolic acids [277] that may explain its favorable effects in experimental studies possibly mediated by antioxidant activities, improvements in microcirculation, modulation of neurotransmitters, enhancement of neuroplasticity, prevention of brain edema, and other neuroprotective actions [268,278,279,280,281,282,283].

*Ginkgo biloba* extracts have been used empirically in the treatment of dementia in humans for several decades [277,284]. However, whether Gb can improve cognitive function and prevent cognitive decline and dementia in clinical studies is as yet a controversial question. Even if few available large-scale clinical trials suggest that Gb extracts are relatively efficacious in delaying the progress of dementia [285,286,287,288], other trials showed negative results [289,290]. Some systematic reviews and meta-analyses have been conducted to assess Gb extracts effects on the prevention and treatment of MCI and dementia [270,291,292,293,294,295,296,297,298,299,300] with mixed results. The available studies are very heterogeneous, but in general, those carried out in healthy people are more frequently negative than those involving patients with MCI or dementia, and the effects seem to be dose-dependent with better results for those using doses of Gb extracts higher than 240 mg/d for longer periods of time. In any case, the results are highly variable, and so far, no definitive conclusions can be made for the use of Gb in cognitive disorders or in the prevention of dementia.

### 3.14. Resveratrol

This is a phytoalexin from the group of polyphenols that is contained in berries. Most of the resveratrol that is consumed through the diet in humans derives from grapes and red wine [301]. Resveratrol as other polyphenols has shown antioxidant and anti-inflammatory actions [302]. Studies conducted in experimental models of AD reporting reduced hippocampal neurodegeneration and improved cognitive memory performance with resveratrol administration [303,304,305,306] provided some optimism to the possibility that this polyphenol could contribute to the prevention of cognitive decline and dementia. Clinical trials in humans are small, fewer, and have shown mixed results [307,308,309,310]. Some small trials exploring intermediate outcomes have reported seemingly beneficial results. For example, a placebo-control trial involving twenty-two healthy adults reported a dose-dependent (250–500 mg/d) increase in cerebral blood flow in the prefrontral cortex during cognitive tasks [311]. Likewise, another small study including twenty-three overweight participants, aged 50–75 years, randomized to receive 200 mg/d of resveratrol or placebo for 26 weeks, reported improved memory performance and higher functional connectivity of the hippocampus in neuroimaging in those participants with resveratrol treatment [312]. However, a recent systematic review and meta-analysis of human clinical trials and animal studies published prior to January 2020 showed that most publications on animal models reported positive outcomes on cognition and brain function following exposure to resveratrol or grape seed extracts. Conversely, eleven meta-analyses of data from human placebo vs. resveratrol, grape, or wine treatment trials did not find any statistically significant effect on various measures, including cognitive performance and mood assessments, gray matter volume, and blood pressure [313].

In summary, the promising effects of resveratrol on cognition and dementia prevention in experimental models are not replicated in human clinical trials based on currently available data. Likewise, as mentioned above (Section 3.9), there is no evidence on the effects of light wine consumption in this regard, while heavy alcohol consumption can be detrimental for brain health [211,222,224]. Therefore, there is no validated evidence for prescribing this supplement or advice recommending wine consumption to improve cognitive function or prevent AD and other types of dementia.

### 3.15. Phytoestrogens

These are plant-derived naturally occurring polyphenolic non-esteroidal xenoestrogens that, because of its structural similarity with estradiol, have the ability to cause estrogenic and/or anti-estrogenic effects by binding to the estrogen receptors. Phytoestrogens have been described in over 300 plants [314]. Isoflavones are the most extensively investigated phytoestrogens, and the neuroprotective effects of soy isoflavones have been demonstrated in experimental animal research and cell cultures studies, which are attributable to both their antioxidant properties and their interaction with the estrogen receptor [314,315,316]. However, the studies include mixtures of them, such as genistein and daidzein, which prevents the identification of which component the cognitive effects depend on. Nevertheless, the studies carried out on soy consumption and phytoestrogen supplements on cognitive function in rodent models and in vitro have shown variable and inconclusive results, most reporting positive neuronal effects with the consumption of soy isoflavores, but high doses may have negative brain and cellular effects [314,315] with an overall absence of adverse events [317].

Some clinical studies reported cognitive positive effects in adult women that were reversed in older women, while in men, the results were even more ambivalent [315]. The inconsistencies found have been explained by the use of diverse isoflavone supplements at various doses in short-term clinical trials. Most studies reporting no effects of phytoestrogens on cognition have been conducted in European cohorts, which is a population with recognized low consumption of soy and soy products. Conversely, studies carried out in Asian populations, in which the consumption of soy-derived products is higher, a lower incidence of cognitive decline associated with this consumption has been observed in older women. The phytoestrogen daidzein is transformed into S-equol by the gut microbiota after oral intake of conjugated isoflavones. However, there are important differences in S-equol production among Japanese and European women, with about 70% of Japanese persons being “equol producers” [318], which can help explain the differences in the results of studies carried out in Japan and in European countries. The Women’s Isoflavone Soy Health (WISH) trial, which was conducted in the USA and involved 313 postmenopausal women aged 45 to 92 years, randomized participants to receive 25 g/d of isoflavone-rich soy protein (containing genistein, daidzein, and glycitein) vs. milk protein as placebo, and evaluated cognitive function at baseline and after two and a half years. The authors did not observe any difference between the groups in the cognitive tests. However, women within five to ten years of menopause in the isoflavone group exhibited a non-significant trend (*p* = 0.07) toward cognitive improvement and a significant enhancement of verbal episodic memory. In addition, participants who were consistent producers of urinary S-equol had a non-significant trend (*p* = 0.08) toward cognitive improvement [319].

In conclusion, the data regarding the beneficial effects of phytoestrogens on cognition appear to be inconclusive. Further well-controlled, large-scale clinical studies are necessary to translate the neuroprotective effects of phytoestrogens reported in experimental models and cell cultures. There are no RCTs of phytoestrogens for the prevention or treatment of AD.

### 3.16. Vitamins

There are numerous studies in the literature investigating possible effects of vitamin supplements intake on cognitive function and on the prevention of cognitive decline and AD, although there are no definitive conclusions. For example, analyses of data from the Physicians’ Health Study and Physicians’ Health Study II (*n* = 4052) reported no significant effects on cognitive function with beta-carotene (provitamin A) treatment in the short-term and beneficial effect with longer-term (18-year) administration [320]. In general, studies of vitamin B on cognition have shown mixed results. In an RCT including 299 men aged over 75 years, no significant effects on cognitive functions were reported after 2-year supplementation with folic acid, vitamin B12, and B6 [321]. Likewise, a meta-analysis including data from nine RCTs (*n* = 2835) found no significant effects of folic acid supplementation with or without other B vitamins on cognitive function [322]. Contrariwise, an RCT involving 900 participants aged 60 to 74 years reported that those receiving a combination of folic acid and vitamin B12 had a significant improvement in cognitive tests when compared to placebo [323]. More recently, a population-based cohort study using Danish registry data assessed possible associations among low plasma vitamin B12 levels, high-dose injection or oral vitamin B12 treatment, and risk of dementia from 2000 to 2013. For the analyses, patients with low plasma B12 levels (<200 pmol/L) were propensity score-matched 1:1 with patients with normal levels (200–600 pmol/L). No associations were found for low plasma B12 levels and dementia, and this was not affected by B12 treatment. Findings were similar for all-cause and vascular dementia. The authors concluded that their results do not support routine screening for B12 deficiency in patients with suspected dementia [324].

A number of studies have investigated the effects of vitamins C and E on cognitive function involving healthy populations with inconsistent results. The Women’s Health Study (*n* = 6377 women over 65 years) reported no significant effects on cognitive function of vitamin E supplementation [325]. Analyses of data from participants of the Duke Established Populations for Epidemiologic Studies (*n* = 616) aged over 65 years found that supplements of vitamins C and/or E did not influence the incidence of dementia or AD [326]. Conversely, data from the Canadian Study of Health and Aging (*n* = 894 participants aged ≥65 years) showed that a combination of vitamins C and E significantly decreased the rate of cognitive decline [327]. One study analyzed brain concentrations of alpha- and gamma-tocopherol in 115 deceased patients with AD from the prospective Rush Memory and Aging Project. Gamma-tocopherol brain levels were associated with lower amyloid load and lower neurofibrillary tangle severity, while alpha-tocopherol levels were not associated with AD neuropathology lesions [328]. Data from the Age-Related Eye Disease Study (*n* = 2166 participants followed for 6.9 years) found that a mixture of anti-oxidant vitamins (vitamins E, C, beta-carotene), zinc, and copper did not have any effect on any of six tests of cognitive function [329]. Likewise, the Women’s Antioxidant Cardiovascular Study involving 2824 women with or at risk for CV disease who received a combination of vitamin E, beta-carotene, and vitamin C or placebo showed no effects on cognition of the multivitamin supplementation [330].

One of the vitamins on which research has remarkably increased in recent years is vitamin D, which has been shown to play various roles in normal brain physiology through its receptor (VDR), which is present in the neurons, glial cells of the hippocampus, orbitofrontal-cortex, cingulate, amygdala, and thalamus [331]. Emerging evidence suggests that low concentrations of vitamin D are potentially involved in the pathogenesis of dementia. However, the underlying mechanisms are still poorly understood. This is of particular interest considering the high prevalence of vitamin D deficiency in older adults and the crucial need to identify modifiable risk factors for dementia. Cross-sectional studies have generally found that vitamin D concentrations are significantly lower in AD patients compared to healthy controls [332]. Some longitudinal cohort studies support an association between low vitamin D concentrations and an increased risk of dementia and cognitive decline [333,334,335,336], although there are also negative results [337,338]. The possibility of reverse causality remains to be answered. Intervention studies have failed to confirm the prevention of cognitive decline and AD with vitamin D supplementation [339,340,341,342,343,344,345,346]. Therefore, although there is certainty that vitamin D is involved in normal brain function and that low vitamin D concentrations can occur in patients with dementia, there is no clear evidence that supplementation with vitamin D can prevent or modify the course of cognitive decline and dementia. The causal relationship between vitamin D and cognitive impairment so far remains inconclusive. Likewise, a recent Cochrane review did not find evidence that any vitamin or mineral supplementation strategy for cognitively healthy adults in mid or late life has a meaningful effect on cognitive decline or dementia, although the authors caution that the available evidence does not permit definitive conclusions [345].

### 3.17. Multinutrient Combination

LipiDiDiet is the first double-blind, multicentre, international RCT of a non-pharmacological intervention in prodromal AD [347,348]. The intervention is the multinutrient combination (Fortasyn Connect) containing DHA, EPA, uridine monophosphate, choline, vitamins B12, B6, C, E, and folic acid, phospholipids, and selenium. These nutrients were selected based on their biological and neuroprotective properties, shown in experimental models and in 3 previous clinical trials of 12-24 weeks treatment, in which improved memory was reported in mild, but not mild-to-moderate AD. LipiDiDiet focused on prodromal AD with a longer duration of the intervention. A first report involving 153 participants treated with the intervention vs. 158 controls found no significant effect on the neuropsychological test battery (NTB) score over two years of intervention, attributable to inadequate statistical power. Group differences on secondary endpoints of disease progression measuring cognition and function and hippocampal atrophy were observed [347]. A second report of data from 162 participants (85 from the active group and 77 from the control group) who completed 36 months of intervention found reduction in NTB decline, Clinical Dementia Rating-Sum of Boxes, memory, and brain atrophy measures [348]. Future studies may assess further benefits by integrating the multinutrient intervention with multidomain interventions such as those discussed below (Section 5).

## 4. Other Non-Dietary Factors That Together with Diet May Influence Cognitive Decline

### 4.1. Physical Activity

Whilst dementia is a common condition in older people, no definitive treatments for this condition are available. Recent works have pointed out that about 3% of all dementia cases could be prevented by increasing levels of physical activity [18,19]. At the same time, increasing literature shows the importance of physical activity and exercise for preventing/slowing down the pathological process and dementia-related problems [20].

It is widely known that older people who are more physically active can maintain cognition for a longer time than sedentary people [18]. In a large meta-analysis, including several cohort studies and 33,816 individuals, higher physical activity levels were associated with a significant reduction in the onset of dementia in a linear dose–response manner [349]. At the same time, the effect of physical activity/exercise in MCI or in early phases of dementia should be better explored. Some studies, in fact, have reported that physical activity/exercise can delay the transition from MCI to dementia [350], but other recent investigations have reported that a moderate-to-high intensity multi-component exercise program is not beneficial in people with early dementia [351].

In our opinion, this topic is important, since MCI is one of the most diffused risk factors for dementia, with 10% to 15% of people with MCI becoming demented during one year of observation [352,353]. Again, whilst several risk factors associated with a faster conversion from MCI to dementia are already known, the research regarding sedentary behavior and physical activity is still limited to a few investigations [354]. In one umbrella review of our group, we have reported that physical activity/exercise was able to significantly improve global cognition and specific cognitive tests in MCI, even if supported by some biases, suggesting a positive role of these interventions, but studies exploring the conversion to dementia are still not present [354].

In people already affected by dementia, it seems that physical activity and exercise can improve global cognition. This finding is probably due to several pathways. First, physical activity/exercise improves the management of cardiovascular risk factors (e.g., diabetes, hypertension, dyslipidemia, and obesity), which are traditionally associated to poor cognitive performance [355]. Furthermore, it is reported that physical activity/exercise may increase neurogenesis and synaptic plasticity in animal models [350,356]. Aerobic exercise is also associated with an increase in brain-derived neurotrophic factor (BDNF), which is a factor that can stimulate neuronal cell growth and can maintain neurons in an optimal status [357]. Finally, using neuroimaging techniques, additional evidence for the impact of physical activity on brain function and structure is reported in human beings [358,359,360]. Physical activity/exercise might be a good predictor of long-term changes in brain structure, such as brain volume [361].

Moreover, in people affected by dementia, physical activity/exercise interventions may have other effects such as decreasing the risk of disability, falls, and neuropsychiatric symptoms [354]. Again, we believe that all these outcomes are important. For example, in one study, it was reported that the incidence of falls in dementia is extremely higher and associated more frequently than controls to fractures and hypokinetic syndrome [362]. Physical activity/exercise may decrease the risk of falls by approximately 31% (204 falls every 1000 people affected by dementia treated with this intervention). Since falls are among the most important contributors of disability, we may hypothesize that the beneficial effect of physical activity/exercise in decreasing the risk of falls may consequently improve activities of daily living [363]. Moreover, another important consequence of physical activity/exercise is to decrease the presence and the severity of neuropsychiatric symptoms [364], in particular depression [365]. Again, it seems that physical activity/exercise may increase the production of neurotransmitters, neurotrophins, and BDNF as well as lead to a reduction of oxidative stress and inflammatory levels, increase cerebral blood flow, regulate the hypothalamic–pituitary–adrenal axis, and support neurogenesis and synaptogenesis [366]. Table 2 shows the summary of systematic reviews and meta-analyses exploring the association of physical activity with cognitive decline and/or incident dementia.

In conclusion, physical activity and exercise are good options for improving not only cognitive aspects but also non-cognitive outcomes in people with MCI and dementia. However, the literature is still sparse, and often, the combination with other non-pharmacological interventions, such as diet, is still limited. In this sense, we warmly suggest future intervention studies that better account for healthy lifestyle in the treatment and prevention of MCI and dementia.

### 4.2. Sleep Pattern

There appears to be a bidirectional relationship between sleep pattern and dementia, with disturbed sleeping representing both a risk factor for, and symptom of, the neurocognitive syndrome [394,395,396]. Indeed, the notion that sleep quality and duration is critical for cognitive processing has gained increasing attention. However, it is not simple to establish causal relations, because there are vicious circles among different aspects of the disease. It is currently accepted that sleep is essential to consolidate memory and to remove the excess of beta-amyloid and hyperphosphorylated tau accumulated in AD patients’ brains [397]. Sleep disturbances frequently precede AD pathological traits and cognitive decline [398]. It has been proposed that sleep alterations (i.e., specific oscillatory patterns) may be biomarkers to predict the risk of developing AD [394]. A systematic review and meta-analysis found that sleep disturbances, including both short and long sleep duration, insomnia, obstructive sleep apnea (OSA), impaired circadian rhythm, and sleep quality, were all associated with an increased relative risk of preclinical AD, cognitive impairment, and AD [399].

Former studies found that sleep disturbances occur frequently in AD and other forms of dementia [400,401]. Recent epidemiological investigations have shown that sleep disorders in AD patients go far beyond the physiological changes that occur in normal aging [399]. As pointed out in a meta-analysis including 5634 AD patients, the prevalence was widely variable, estimated in 14–69% [402]. A retrospective study conducted in Japan involving 684 AD patients found a prevalence of 21.3% [403]. Other studies reported that over 60% of patients with MCI and AD had at least one clinical sleep disorder, of which insomnia and OSA were the most common [404,405]. The inconsistent prevalence reported may be related to the use of sleep questionnaires in many studies; understandably, cognitive impairment in patients with AD renders the information on sleep disturbances provided by a questionnaire not fully reliable. It has been reported that patients with AD can have increased sleep onset latency and reduced time spent in restorative slow wave sleep and rapid eye movement sleep [406]. In dementia with Lewy bodies, sleep disturbance may be even more common than in AD, and they have been associated with rapid eye movement sleep behavior disorder [407]. A relevant point is that sleep disturbances may significantly impact caregiver burden, caregiver sleep, and quality of life, and it is associated with a higher risk of institutionalization [408,409].

Various medications are commonly used for sleep disturbance in dementia, but evidence is lacking for most of them [410]. Melatonin may be beneficial in some cases, and it may help for nocturnal behavioral and psychological symptoms of dementia (BPSD), together with non-pharmacological interventions, which should be first-line therapy [411,412]. Trazadone may be effective for sleep disturbance and BPSD, in case the pharmacological intervention is considered necessary [413]. Indeed, non-pharmacological management has an important role due to the risk of adverse effects associated with the use of hypnotics [414]. A systematic review and meta-analysis [415] explored non-pharmacological treatments for sleep disturbance in MCI and dementia including 48 articles with participants aged 67.3 to 89.4 years. Most studies (79%) had small samples (less than 50 participants), and many were conducted in long-term care settings (62%). Most recruited participants had moderate–severe dementia (85%) with a wide range in MMSE scores (0 to 28.3/30), while only four studies examined MCI. Light therapy for one to ten weeks was the most frequently studied intervention. with most studies (81.5%) showing improvements on objective or subjective sleep measures, although with significant clinical and methodological heterogeneity that prevented a definitive conclusion. There were seven multi-modal intervention studies, all incorporating light exposure, and six of them reported improved sleep. Other less frequently used interventions included electrotherapy stimulation, physical exercises/activities, acupressure/acupuncture, and mindfulness/cognitive behavioral therapy. The authors performed a meta-analysis of data from RCTs reporting a statistically significant improvement in sleep efficiency between multi-modal interventions and controls, favoring the combined interventions (bright light, multidomain, and other therapies) [415]. Therefore, non-pharmacological sleep interventions seem useful, particularly multidomain approaches, but the available studies are heterogeneous and small. More research evaluating the efficacy of multimodal interventions in community-dwellers and those with MCI is still needed.

### 4.3. Social Engagement

Social engagement emerged about two decades ago as a potential protective factor against cognitive decline and dementia [416,417]. Afterwards, other evidence appeared confirming these findings [418,419,420]. Likewise, late-life leisure activities involving physical, mental, and social domains have been reported to be key contributors to brain protection [421,422]. A meta-analysis comprising data from nineteen longitudinal studies up to July 2012 found that low social participation, greater loneliness, and less frequent social contact were all associated with an increased risk of dementia [423]. A more recent meta-analysis [424] including 31 cohort and two case-control studies comprising 2,370,452 participants found that poor social engagement indices were associated with increased dementia risk, including having a poor social network and poor social support. In long-term studies (≥10 years), good social engagement was modestly protective, with moderate heterogeneity and significant publication bias for this outcome. Loneliness showed a non-significant trend of association with increased risk without substantial heterogeneity or publication bias. However, only four studies could be included, and of these, only one examined loneliness with a validated scale, with the remaining studies relying on a single question or a few questions in questionnaires. In subgroup analyses, studies adjusted for depression showed that overall, poor social engagement was associated with an even greater (56%) increased risk of dementia with no heterogeneity or publication bias. Likewise, when considering only the highest quality studies, the association between poor social engagement and dementia risk was maintained at approximately 55% increased risk, with only mild heterogeneity [424]. This is strong evidence for poor social engagement as a risk factor for dementia, which encourages interventions targeting social isolation and disengagement for dementia prevention.

Various neurobiological mechanisms have been suggested to help explain the direct or indirect association of social engagement and dementia risk [416]. One of them is the stress hypothesis, in which a hypersecretion of glucocorticoids in the stress response causes accelerated hippocampal degeneration with a consequent tendency to cognitive decline or dementia [425]. Another hypothesis states that that social engagement attenuates the risk of neurodegenerative disease by minimizing cardiovascular risk factors. Nevertheless, the best supporting evidence exists for a third hypothesis, which postulates that neuropathological and atrophic changes that may manifest as cognitive deficits in some people may not cause symptoms in others who have higher cognitive reserve [426,427,428]. This was convincingly demonstrated in the Nun Study, in which about one-third of cognitively normal nuns were found to have high levels of neuropathology consistent with AD at autopsy [419,429]. Similarly, in the Rush Memory and Aging Study, in those with more extensive social networks, more extensive neuropathological lesions were associated with less deleterious changes in working and semantic memory compared to those with limited social networks [420]. A recent meta-analysis conducted in nine studies evaluating the effect of cognitive reserve on progression to MCI or dementia showed that high cognitive reserve was related to a 47% reduced relative risk of MCI or dementia. Considering residual-based cognitive reserve (cognitive residual of neuropathology and as composite psychosocial proxies, i.e., education), the risk was further reduced (62% and 48%, respectively) [428]. These results strongly support that cognitive reserve protects against MCI and dementia progression above and beyond the effect of AD-related structural pathology and biomarkers. Recent data confirmed the cognitive reverse model. A prospective study analyzed data from 2099 participants aged 65 or over from the Cognitive Function and Ageing Study—Wales (CFAS-Wales). Dementia was ascertained through the comprehensive judgment symptoms of geriatric mental state automated geriatric examination for computer-assisted taxonomy (GMS-AGECAT). Subjective cognitive decline was evaluated by two questions in the baseline interview. Cognitive reserve indicators were derived from three previously identified factors: early life education, midlife occupational complexity, and late-life cognitive activities. Baseline subjective cognitive decline and low cognitive reserve significantly increased the risk of dementia after two years of follow-up. Statistically significant association between subjective cognitive decline and dementia was found only in the low- and medium-level cognitive reserve group, indicating that cognitive reserve attenuated subjective cognitive decline-associated risk of developing dementia [430]. Recent analyses of data from the Rush Memory and Aging Project, involving 1697 dementia-free participants at baseline (mean age: 79.6 years) followed up to 21 years, found that the highest cognitive reserve was related to a slower decline in global cognition, episodic memory, and working memory compared to the lowest in multi-adjusted mixed-effect models. This association remained significant among participants with high AD pathology or gross infarcts [431]. Another recent retrospective cross-sectional study using data from 2171 participants of the Framingham Study (mean age 63 ± 10 years) examined the association of individual forms of social support with a global neuroanatomical measure of early AD vulnerability and cognition. Participants who were free of dementia, stroke, or other neurological conditions at baseline underwent brain magnetic resonance imaging and neuropsychological testing. Cognitive resilience was defined as the modification of total cerebral volume’s association with cognition. The results showed that social support in the form of supportive listening was associated with greater cognitive resilience, independently modifying the association between lower total cerebral volume and poorer cognitive function that would otherwise indicate increased AD vulnerability at the preclinical stage [432].

Thus, these studies support potential links between socialization and cognitive reserve. However, a limitation in these studies is reverse causality: dementia could lead to social isolation and social withdrawal because prodromal and subclinical signs and symptoms of dementia may be present even a decade before its onset. Some studies have attempted to address this issue by excluding incident dementia cases early in the observational period, but longitudinal studies with longer follow-ups are still needed to clarify the possibility of reverse causation.

Erikson’s model developed in the 1950s proposed that adult psychosocial development outlines the significance of successful involvement within one’s relationships, work, and community for healthy aging. Data from participants of the Study of Adult Development including 159 over-75-year-old men from both higher and lower socioeconomic strata evaluated whether late-life depression mediated the relationship between Eriksonian development and specific domains of cognitive functioning (i.e., executive functioning and memory). The results indicated that higher midlife Eriksonian psychosocial development at ages 30–47 was associated with stronger global cognitive functioning and executive functioning and lower levels of depression three to four decades later (ages 75–85) with no significant association between Eriksonian development and late-life memory. Late-life depression mediated the relationship between Eriksonian development and both global cognition and executive functioning. These findings have important implications for understanding the lasting benefits of psychosocial engagement in mid-adulthood for late-life cognitive and emotional health. Furthermore, it may be that less successful psychosocial development increases levels of depression, making individuals more vulnerable to specific areas of cognitive decline [433].

In summary, while understanding that no definitive statements about causation can be made, interventions to improve poor social engagement may potentially reduce incident dementia. This potential warrants well-designed trials including investigation of potential neurobiological, behavioral, and social mechanisms to validate the plausibility of the cognitive reserve hypothesis generated by observational cohort studies.

## 5. Multidomain lifestyle-based interventions

Due to the complex, multifactorial, and heterogeneous nature of age-related cognitive decline and of late-onset AD and dementia, it is unlikely that a single intervention can have a significant impact. A crucial consideration regarding preventive interventions is the fact that multiple risk and protective factors for cognitive decline and dementia usually coexist and interact across the lifespan to determine the overall risk of dementia. Therefore, interventions targeting several risk factors and mechanisms simultaneously may be required for optimal preventive effects. Up to now, the results of three large multidomain lifestyle-based prevention trials have been published: the Finnish Geriatric Intervention Study to Prevent Cognitive Impairment and Disability (FINGER) [10], the French Multidomain Alzheimer Preventive Trial (MAPT) [243], and the Dutch Prevention of Dementia by Intensive Vascular Care (PreDIVA) [434].

The Finnish Geriatric Intervention Study to Prevent Cognitive Impairment and Disability (FINGER) is the first large, long-term RCT to demonstrate that a two-year multidomain lifestyle-based intervention by improving vascular and lifestyle-related risk factors can preserve cognitive performance and reduce the risk of cognitive decline among older adults at increased risk of dementia [10]. The FINGER RCT comprised 1260 older adults aged 60-77 years; the multidomain intervention delivered by trained professionals through individual and group sessions consisted of dietary counseling, exercise, cognitive training, social activities, and monitoring and management of vascular and metabolic risk factors. The control group was offered regular health advice. After two years, the intervention showed significant beneficial effects on the neuropsychological test battery composite score (25% more improvement vs. control), executive functioning (83% more improvement), processing speed (150% more improvement), and complex memory tasks (40% more improvement) [10]. There were also favorable effects on BMI reduction, improved adherence to dietary guidelines and recommendations [435], and increase in physical activity [10].

The other two RCTs, MAPT [243] and PreDIVA [434], reported negative results for their primary outcomes although subsequent subgroup analyses showed some benefit. The MAPT RCT enrolled 1680 community dwellers aged 70 years or older who had either subjective memory complaints, limitation in one IADL, or slow gait speed. In two arms, participants received a multidomain lifestyle intervention consisting in cognitive training and counseling on nutrition and physical activity, either alone or in combination with omega-3 fatty acid supplementation. In other two arms, participants received only the omega-3 fatty acid supplementation or placebo. The primary outcome was change in a cognitive composite score. Although the trial failed to meet its primary outcome, beneficial intervention effects were observed when both groups receiving the multidomain lifestyle intervention were combined. Beneficial effects were also reported for specific subgroups: those with brain amyloid pathology or severe cognitive impairment (CAIDE risk score ≥6 points) [243,436]. The PreDIVA trial enrolled 3526 unselected older adults aged 70-78 years from general practices [434]. The multidomain intervention consisted of advice concerning healthy lifestyle and intensive vascular care and risk factor management, including initiation or optimization of antithrombotics and pharmacological treatments for hypertension, diabetes, or dyslipidemia, when necessary vs. regular care for the control group. After six years of intervention, there was no difference in the primary outcome (i.e., dementia incidence) between the intervention and control groups. However, in the exploratory analyses, a reduction in the incidence of dementia was observed among participants with untreated hypertension who adhered to the treatment during the trial.

Several issues may help to explain the dissimilar results of the last two trials, including the difference in the selection of the studied populations; also the fact that the intervention should start early enough to reduce the long-term exposure to the risk factors and should have a long-term follow-up to make evident any benefit for a robust outcome such as dementia.

Innovative proposals of multidomain prevention trials have started to use novel technologies, such as eHealth and mHealth, to optimize the delivery of multidomain interventions. For example, the Healthy Aging Through Internet Counselling in the Elderly (HATICE), a European open-label 18-month RCT testing the efficacy of an Internet platform in improving self-management of CV risk factors for prevention of CV disease and cognitive decline in over-65 participants reported a modest but significant improvement in CV risk profiles with the interactive Internet intervention after 18 months [437]. If preventive interventions through Internet or via mobile applications prove to be feasible and effective and induce sustained behavioral changes on a large scale, it could support self-management and be a cost-effective way to reach and involve a large population across the world.

Following the encouraging results of the FINGER trial, the World-Wide (WW)-FINGERS network was launched in July 2017 [438]. By collectively convoking international research teams, WW-FINGERS aims to facilitate data sharing and joint analyses of studies across country borders, and strengthen the potential evidence-base for multidomain lifestyle interventions worldwide in at-risk populations from diverse geographical and cultural settings, by means of local and cultural adaptations of content and delivery method of interventions. For example, it is proposed that dietary counseling will follow national recommendations and take into account country- or region-specific habits, and pharmacological CV risk factor management based on national care guidelines. Several countries worldwide have joined the WW-FINGERS network and are currently at different stages of planning and conducting their FINGER-type prevention trials [438].

## 6. Proposed Mechanisms Mediating the Effects of Nutrition and Other Lifestyle Factors on Cognitive Decline

### 6.1. Oxidative Stress and Chronic Inflammation

Aging and age-related degenerative diseases have been linked to a pro-oxidant and pro-inflammatory state, which leads to the damage of cellular components. The brain tissue is highly susceptible to oxidative damage because cerebral metabolism necessitates large quantities of energy, is dependent on aerobic conditions, and is rich in oxidizable compounds such as PUFAs and in transition metals that facilitate free radicals generation. Furthermore, compared to other body structures, the brain has low concentrations of antioxidant systems. This may help to explain why the brain tissue is so susceptible to damage due to an accumulation of neurotoxic peptides such as amyloid-beta [439]. In studies performed in autopsies from AD patients, brains exhibited increased protein oxidative damage, glycol-oxidation, lipid peroxidation, and reduced antioxidant enzyme systems [440].

There is growing evidence linking neuroinflammation to AD pathogenesis. Misfolded and aggregated proteins, such as amyloid, bind to toll-like receptors (TLRs) and CD4 in the microglia, starting innate immune responses with the production and release of inflammatory mediators [441,442]. Alois Alzheimer already had described the microglia surrounding amyloid plaques and tangles describing the disease for the first time, but the key role of microglia inflammation has been investigated only recently. In normal conditions, neuroinflammation is a crucial protective mechanism. Conversely, when it becomes excessive, chronic, and uncontrolled, it may become damaging by the continuous release of cytokines, proteolytic enzymes, free radicals, complementary factors, nitric oxide, or excitatory amino acids [442,443]. Amyloid-beta accumulation further induces neuroinflammation, which produces more amyloid-beta aggregation, leading to a vicious cycle of propagating injury [444]. Increased neuroinflammation has been shown in MCI and AD patients vs. healthy controls using positron emission tomography and imaging with radioligand C-11-DAA1106 [445]. Amyloid-beta may promote a preponderance of macrophage (M)1 pro-inflammatory cells vs. M2 (anti-inflammatory cells) and decrease the switching of cellular phenotypes to lessen destruction [442,443]. Excess of nitric oxide may also contribute to induce inflammatory signals, which are key players in neurodegenerative diseases with resulting neuronal death [446].

### 6.2. Overweight and Obesity

The well-known CV and metabolic risk factors, both in isolation and combined, have been recognized as risk factors for cognitive decline and AD as well [18,447]. The metabolic syndrome—namely, the simultaneous occurrence of diverse risk factors (i.e., central obesity, hypertension, hyperglycemia, dyslipidemia, and prothrombotic state)—is directly related to increased visceral adiposity in midlife as a consequence of overeating and sedentary lifestyle with harmful consequences in late life [448]. Obesity is currently a pandemic at all ages [449,450,451]. Currently, there is a large availability of inexpensive processed food, full of calories and poor in nutrients, which together with little physical activity have paradoxically contributed to the increased life expectancy in the last century. Overeating and a sedentary lifestyle are considered powerful risk factors for the genesis of chronic non-communicable diseases, which are becoming unsustainable [452]. A systematic review of twenty-eight cohort studies conducted from 2003 to 2013 with a follow-up range between five and forty years reported up to a 2.44-fold increased risk of late-onset dementia in participants who were overweight and obese in midlife [453].

Although the precise mechanism behind this association is not yet completely clear, various possible mechanisms have been suggested. Overweight and obesity are strong risk factors for cardiometabolic disease (i.e., hypertension, diabetes, and dyslipidemia), which are also recognized risk factors for dementia [18,447]. However, these factors have been taken into account in most studies as potential confounders. Obese persons with diabetes had higher concentrations of plasma amyloid proteins [454], and those with history of being overweight or obese in midlife had lower blood–brain barrier integrity after twenty-five years of follow-up [455]. Another source of inflammation that may trigger systemic and neuroinflammation lies in the accumulation and activation of macrophages around the excess fat cells that accompany obesity. These immune cells present in adipose tissue can produce pro-inflammatory cytokines (e.g., interleukin (IL)-1b, IL-6, tumor necrosis factor –TNF-) as well as less anti-inflammatory cytokines (e.g., adiponectin and IL-10), which sustain the state of low-grade chronic inflammation [456]. Obesity has been also associated with a switch from M2 macrophages to the pro-inflammatory M1 phenotype [442,457].

Lipids contained in the diet, and more so saturated fatty acids, induce inflammatory responses on microglia, with local cytokine secretion, i.e., hypothalamic nuclear factor kappa B (NF-kB), which may promote the apoptosis of neurons linked to body weight control, glucose homeostasis, central regulation of energy balance, and blood pressure [458,459]. Obesity may induce alterations in the developing brain of children and adolescents [460], with long-term adverse consequences. Contrariwise, weight reduction, low-calorie diets, and frequent consumption of food rich in anti-oxidant/anti-inflammatory properties, or food patterns with combinations of them, have been associated with reduced levels of systemic and adipose tissue inflammation markers [461]. Clinical studies have shown a link between insulin resistance and cognition [462] as well as between glucose regulation abnormalities in type 2 diabetes and cognitive function [463]. Accordingly, diet interventions that improved insulin resistance have been associated with decreased inflammatory cytokines concentrations and improved cognition [464,465].

### 6.3. Microbiota

The human gut microbiota comprises trillions of symbiotic microorganisms, which are crucial for immune and brain health and disease [466]. The main component of gut microbiota is bacteria, of which most are strict anaerobes, but it also comprises fungi and viruses. The main reported phyla in gut microbiota from adults are Bacteroidetes and Firmicutes, while Proteobacteria and Actinobacteria are found in relatively low amounts [467]. The number of gut microbes within the gastrointestinal tract is estimated to be approximately 100 trillion, which is 10 times the number of cells found in the human body. The gut microbiome is about 150 times greater than that of the human genome and encodes 100-fold more unique genes than our genome, which suggests their vast possibilities in influencing human health. This microbiome shows vast diversity amongst different population groups and has a significant influence on human health, disease state, and overall well-being [468]. Several factors may alter the composition of gut microbiota, such as diet, antibiotic exposure, and infection, which may promote the loss of homeostasis and have been implicated in the development of various diseases, including obesity, metabolic syndrome, colorectal cancer, type 2 diabetes, allergies, inflammatory bowel disease, heart failure, and neurodegenerative disorders [469,470,471,472]. Recent investigations suggests that the amyloid cascade model does not fully explain AD pathogenesis and that alterations in the gut microbiome may play a significant role in the genesis and progression of the disease [473].

The bilateral interaction of the gastrointestinal tract and the brain was recognized for the first time in the 1880s by William James and Carl Lange, proposing that this gut–brain communication could play a role in emotional regulation. At present, the microbiota has been added to this connection (microbiota–gut–brain axis), and it is believed to constitute a two-way homeostatic pathway, through which the gastrointestinal tract exerts modifications on brain function and vice versa [474,475,476].

This microbiota–gut–brain axis bidirectional communication occurs through various pathways. (1) First, over thirty hormones and neurotransmitters are produced in the gastrointestinal tract. These hormones affect the brain centers that regulate metabolic control, appetite, and behavioral pathways linked to reward, anxiety, mood, memory, stress, etc. They can also act locally and activate afferent vagal terminals in the gut, thus generating afferent signals [474,475,477]. Through entero-endocrine cells, enteric neurons, and gut microbiota, the gastrointestinal tract can as well synthesize neurotransmitters that influence the functioning of both the gastrointestinal tract and the central nervous system (CNS) [475]. Neurotransmitters that impact several brain functions, including cognition (i.e., serotonin, dopamine, acetylcholine, gamma-aminobutyric acid, epinephrine, and norepinephrine), can all be synthesized in the gastrointestinal tract [478]. (2) In neuroanatomic communication, the main neuroanatomical communication between the enteric nervous system and the CNS is provided by the vagus nerve (parasympathetic input) and spinal nerves (sympathetic input). Interestingly, 90% of vagal fibers are afferent, suggesting that the brain is primarily a receiver of information, rather than a transmitter, with regard to gut–brain communication [479]. The vagus nerve has receptors for some hormones and neurotransmitters produced in the gastrointestinal tract, such as serotonin, ghrelin, cholecystokinin, and YY peptide, as well as receptors for bacterial fragments, such as lipopolisaccaride (LPS), whose activation can signal the brain regarding gut events. Furthermore, short-chain fatty acids (SCFAs) produced by the gut microbiota can also activate afferent fibers of the vagus nerve [480]. In support of this bidirectional communication, vagotomy has recently been shown to prevent the beneficial neurobehavioral effects induced by probiotics [481]. (3) Activation of the hypothalamic–pituitary–adrenal (HPA) axis leads to the release of cortisol by the adrenal gland during acute stress. In normal conditions, the neuroendocrine stress response is counter-regulated by a negative feedback mechanism. However, if stress is exaggerated and/or persistent, excess cortisol can be deleterious [482]. Stress can cause increased gastrointestinal motility, secretions, permeability, and also adverse effects on the gut microbiota [483]. Contrariwise, changes in microbiota can also modify the stress response. A study showed that germ-free animals had an exaggerated HPA stress response compared to animals colonized with beneficial bacteria [484]. Another preclinical study demonstrated that microbiota can modulate the stress-dependent activation of pituitary and adrenal glands [485]. It is well recognized that chronic stress can affect the brain (and in particular hippocampal) functioning, including learning and memory processes, as well as mood regulation [482,486]. (4) Metabolites produced by bacteria, such as SCFAs, including acetate, propionate, and butyrate, can also influence the gut microbiota–gut–brain crosstalk [487]. SCFAs are produced by bacterial fermentation of substrate (mostly dietary fiber) in the large intestine. The functions of SCFAs are not yet totally clarified, but it has been suggested that they may have local effects in the colon (e.g., decrease inflammation, improve mucus production), affect gene expression by inhibiting histone deacetylases, impact hormone regulation (e.g., glucagon-like peptide 1 and peptide YY), and interact with vagal afferents. Furthermore, SCFAs can also regulate blood–brain barrier integrity and function, acting directly on the brain [488]. (5) The gastrointestinal tract contains about 70% to 80% of the body’s immune cells [489]. Components of Gram-negative bacteria such as LPS can interact with TLRs, influencing the immune response and the production of inflammatory cytokines [490], which can reach or send signals to the CNS through several mechanisms and result in neuroinflammation [471,491]. Moreover, the CNS regulates innate immune responses through neuronal and hormonal routes [492]. (6) BDNF is involved in synaptic and structural plasticity, learning, and memory, and it has been associated with the pathophysiology and treatment of several neurological diseases [493]. A preclinical study showed that germ-free mice had reduced BDNF expression in the cerebral cortex and hippocampus compared to controls [484]. Germ-free mice had lower BDNF mRNA expression in the hippocampus, cingulate cortex, and amygdala [494], while animals supplemented with probiotics or prebiotics showed increased BDNF levels in various brain regions [495]. (7) A number of intrinsic and extrinsic factors can influence the microbiota–gut–brain axis. These include genetic and epigenetic factors, as well as environmental factors, such as exercise, medications, and consumption of probiotics [476]. Diet composition and nutritional status have repeatedly been shown to be crucial modifiable factors regulating the gut microbiota across the lifespan and under various health conditions [496].

Most investigations on host–microbiota interactions come from animal models, which represent crucial tools for studying the various pathways linking the gut and the brain. Nevertheless, there are complexities and marked limitations in translating complex human disease to reductionist animal models. Studies in humans are few and small. For example, several bacteria taxa (i.e., Bacteroides Actinobacteria, Ruminococcus, Lachnospiraceae, and Selenomonadales) were found to be different in forty-three AD patients compared to those in healthy controls using RNA sequencing [497].

In summary, despite the preclinical evidence, well-designed clinical studies are still lacking to elucidate the role of the microbiota or the microbiota–gut–brain axis on the prevention and treatment of cognitive decline.

### 6.4. Autophagy

The efficiency of autophagy declines with age. This crucial mechanism mediates the degradation and recycling of cellular proteins and clearance of misfolded proteins and aggregates such as amyloid, which is one of the most studied pathophysiological mechanisms in AD [498]. The mammalian target of rapamycin (mTOR) signaling, a fundamental player of cellular senescence, affects glucose metabolism, mitochondrial function, energy production, and autophagy in the brain. These events are crucial in age-associated cognitive decline and AD [499].

### 6.5. Prothrombotic State

Vascular disorders are other features characteristic of AD (e.g., cerebrovascular dysfunction, blood–brain barrier disruption, and decreased cerebral blood flow) as well as a prothrombotic state (e.g., activated platelets, clot formation, decreased fibrinolysis). Fibrinogen accumulates with amyloid-beta, which triggers amyloid-beta fibrillization and the generation of fibrin resistant to degradation. A study showed higher platelet activating factor acetylhydrolase activity and higher oxidized-LDL concentrations in AD patients compared to healthy controls [500].

## 7. Summary/Conclusions

The aging of world populations is an undeniable reality that leads to an inexorable increase in aging-related cognitive decline and its worst manifestation, dementia. The accumulation of negative results in the last decades together with the heavy financial burden while searching for effective pharmacological therapies suggest that this is the wrong path. Compelling evidence has accumulated on the critical role that adequate nutrition together with other lifestyle factors can play in the maintenance of cognitive health and in the prevention of cognitive decline and its progression to dementia. It is credible that this must be the path to travel.

However, the illusion that one or few nutritional components can be effective in this complex battle is short-lived. Nutrition research has shifted its focus in recent years from examining the effects of foods or nutrients in isolation to focusing the attention on the effects of foods/nutrients combinations in dietary patterns [17], which the results presented in this review of the field of cognitive function once more corroborate as a wiser choice. In this review, which was more focused on nutrition but without neglecting the other crucial contributing lifestyle factors, it is clear that no single food or nutrient is the magic bullet to prevent dementia, even with some that have shown potential neuroprotective actions, but none with definitive answers as independent determinants of cognitive decline and dementia. Most of these components have been extensively studied in vitro or in experimental animals, but evidence is lacking in studies involving humans because most studies have small samples, short follow-up periods, or have unsuitable designs. On the contrary, it is reasonable to think that the combinations of foods and nutrients, as shown by studies on dietary patterns, such as the MedDiet, DASH, and MIND diets, comprising mostly plant-based unprocessed or minimally processed foods and nutrients that are considered neuroprotective, such as vegetables, EVOO, berries, fruits, and nuts, are the ideal way to counteract the increase in the decline of cognitive functions. Clearly, to this must be added other essential factors such as physical activity, sleep quality, and socialization, among non-dietary determinants. A recent systematic review and Bayesian network meta-analysis aiming to determine and compare the contributions of modifiable risk factors with the prevention of dementia in older adults with analyses of data from forty-three cohort studies defined factors associated with lower risks of all-cause dementia. These factors included no sleep disturbances, a high level of education, no history of diabetes, non-obese patients, no smoking history, living with family members, participation in physical exercise, abstinence from drinking, and no history of hypertension. The findings provide reliable support for the hypothesis that modifiable somatic and lifestyle factors are strong predictors of all-cause dementia [19].

It is reasonable to anticipate that complex diseases come from the combination of a series of factors over time, and accordingly, it is logical to foresee that preventive measures should be similarly combined. It should be learned from the accumulated evidence that the design of future studies should be multi-component from the nutritional viewpoint (combinations of foods and nutrients) jointly with other lifestyle aspects.

Although the mechanisms that may explain the benefit of healthy eating patterns and other lifestyle factors in the prevention of cognitive deterioration are not yet fully clear, studies on the anti-inflammatory and anti-oxidant properties, on the influence on the microbiota, on vascular disorders and prothrombotic states, and on mediators controlling the clearance of misfolded proteins such as amyloid may help explain the observed benefit.

Dementia is particularly feared among people for the possibility of losing self-sufficiency and the need to depend on others. It is frequently perceived as a fatal fate for which there is little to be done to avoid it. Perhaps there is more awareness among the general public of the opportunity to prevent cardiovascular disease but less understanding of the fact that dementia can be largely prevented with a long-life proper nutrition and healthy lifestyle. Promoting this knowledge can be viewed as an opportunity to persuade people in adhering to conscious and healthy food and lifestyle choices leading to an active and healthier aging.

## Figures and Tables

**Table 2:** Summary of systematic reviews and meta-analyses exploring the association of physical activity with cognitive decline and/or incident dementia.

First Author of the Review	Year	Type of Review	Total Sample Size	Physical Activity/Exercise Description (Type, Frequency, Duration, Intensity) Intervention	Summary of Results
Adamson [367]	2015	MA	1324	Physical activity mixed. Mean frequency 3 times per week; Mean duration 40 min	Physical exercise, particularly when meeting physical activity guidelines, can improve depressive symptoms in adults with neurologic disorders, with a less evident effect in dementia.
Almeida [368]	2019	MA	1129	Home-based physical activity. No details regarding mean frequency, duration, or intensity	Home-based physical activity seems safe and effective in delaying cognitive function decline and improving changes in behavioral and psychological symptoms of dementia, activities of daily living, health-related physical fitness, and carer’s burden in people with dementia living at home.
Barreto [369]	2015	MA	1627	Physical activity mixed. Mean frequency 3.38 times per week; Mean duration 38 min	Physical exercise reduces depression levels in dementia.
Brown [370]	2019	MA	524	Home based physical activity. No details regarding mean frequency, duration, or intensity	The effectiveness of home-based exercise programs on mobility in community-dwelling people with Alzheimer’s disease remains inconclusive.
Burge [371]	2012	MA	278	Physical activity mixed. Mean frequency 2.8 times per week; Mean duration 36.6 min	Physical exercise may limit the negative effects of dementia in activities of daily living.
Burton [372]	2015	MA	336	Physical activity mixed. No details regarding mean frequency, duration, or intensity	Exercise program may potentially assist in preventing falls of older people with dementia living in the community.
Cammisuli [373]	2017	MA	768	Aerobic exercise. Mean frequency 2.55 times per week; Mean duration 57 min	Aerobic exercise improves cognition in MCI patients. Overall research reported moderate effects for global cognition, logical memory, inhibitory control, and divided attention.
Cammisulli [374]	2018	MA	554	Aerobic exercise. Mean frequency 2.57 times per week; Mean duration 46.25 min	There is scarce evidence that aerobic exercise improves cognition in AD patients.
Fleiner [375]	2017	MA	201	Physical activity mixed. Mean frequency 3 times per week; Mean duration 56 min	Exercise represents a potentially worthwhile approach for the treatment of patients suffering from BPSD.
Gates [376]	2013	MA	1695	Physical activity mixed. Mean frequency 3 times per week; Mean duration 40 min	Limited evidence that exercise improves cognitive function in individuals with MCI.
Groot [377]	2016	MA	802	Physical activity mixed; Mean duration 146 min	Physical activity interventions positively influence cognitive function in patients with dementia.
Jia [378]	2019	MA	673	Physical activity mixed; Mean frequency 1.8 times per week; Mean duration 40 min	Physical activity and exercise can improve cognition of older adults with AD.
Leng [379]	2018	MA	2289	Physical activity mixed. No details regarding mean frequency, duration, or intensity	Physical exercise significantly ameliorated depressive symptoms and BPSD.
Lewis [380]	2017	MA	945	Home-based physical activity. No details regarding mean frequency, duration, or intensity	Long-term home and community-based exercise programs improve function in older adults living in the community with cognitive impairment.
Li [381]	2019	MA	2051	Physical activity mixed. No details regarding mean frequency, duration, or intensity	Physical exercise programs might play an important role in cognition and ADL in patients with dementia.
Lim [382]	2019	MA	325	Tai Chi. Mean frequency 3 times per week; Mean duration 46 min	Tai Chi may improve short-term cognitive function in older people at the onset of dementia.
Ojagbemi [383]	2019	MA	765	Physical activity mixed. Mean frequency 1 times per week; Mean duration 130 min	Physical exercise interventions led to small and non-significant improvement in quality of life in older people affected by dementia.
Packer [384]	2019	MA	3380	Physical activity mixed. Mean frequency 1.66 times per week; Mean duration 120 min	No clear benefit or harm associated with any of interventions on risks of hospitalization, duration of hospitalization, or death.
Pisani [385]	2021	MA	1793	Physical activity mixed	The effect size for physical exercise was generally comparable to donepezil.
Song [386]	2018	MA	929	Physical activity mixed. Mean frequency 2.45 times per week; Mean duration 54.5 min	Physical exercise, aerobic exercise in particular, benefits global cognition in MCI patients.
Wang [387]	2019	MA	1088	Resistance training; Mean frequency 2.5 times per week; Mean duration 63 min	High-intensity and frequent resistance exercises may be the most effective exercise type to improve global cognition in adults with mild cognitive impairment.
Zheng [388]	2016	MA	1587	Aerobic exercise. Mean frequency 2.28 times per week; Mean duration 53 min	Aerobic exercise led to an improvement in global cognitive ability and had a positive effect with a small effect size on memory in people with MCI.
Zou [389]	2019	MA	940	Mind body exercises. Mean frequency 3.8 times per week; Mean duration 60 min	Mind–body exercise may have the potential to improve various cognitive functions in people with MCI.
Blankevoort [390]	2010	SR	642	Physical activity mixed. No details regarding mean frequency, duration, or intensity	Multi-component interventions can improve physical functioning and ADL in elderly subjects regardless of the stage of dementia. The best results were obtained in the interventions with the largest training volume.
Brett [391]	2016	SR	901	Physical activity mixed. No details regarding mean frequency, duration, or intensity	Significant effect of physical activity on functional ability, particularly on mobility items.
Guitar [392]	2018	SR	259	Physical activity mixed. Mean frequency 2.5 times per week; Mean duration 129 min	Significant improvement was seen on executive function in older people affected by AD.
Learner [393]	2016	SR	423	Physical activity mixed. Mean frequency 3,4 times per week; Mean duration 37.5 min	There is moderate-to-strong evidence that physical activity can effectively maintain cognitive function in nursing home residents with dementia.
Demurtas [354]	2020	UR	28,205	Mixed	Supported by very low-to-moderate certainty of evidence, physical activity/exercise has a positive effect on several cognitive and non-cognitive outcomes in people with MCI and dementia.

AD: Alzheimer’s disease; BPSD: behavioral and psychological symptoms of dementia; MA: meta-analysis; MCI: mild cognitive impairment; SR: systematic review; UR: umbrella review.

## Data Availability

No new data were created or analyzed in this study. Data sharing is not applicable to this article.

## Abbreviations

AD: Alzheimer’s disease; ADAS-cog: Alzheimer’s Disease Assessment Scale-Cognitive subscale; ADCS-ADL: Alzheimer’s Disease Cooperative Study—Activities of Daily Living and Quality of Life in AD; AIBL: Australian Imaging, Biomarkers and Lifestyle; APOE ε4: apolipoprotein E epsilon4; ARIC: Atherosclerosis Risk in Communities; BDNF: brain-derived neurotrophic factor; BPSD: behavioral and psychological symptoms of dementia; BVMT-R: Brief Visuospatial Memory Test—Revised; CAIDE: Cardiovascular Risk Factors, Aging and Dementia; CANTAB PAL: Cambridge Neuropsychological Test Automated Battery Paired Associate Learning; CDB: Cognitive Demand Battery; CFAS-Wales: Cognitive Function and Ageing Study—Wales; CNS: central nervous system; CREB: cyclic amp-response element binding protein; CV: cardiovascular; DASH: Dietary Approaches to Stop Hypertension; DG: dentate gyrus; DHA: docosahexaenoic acid; DSST: Digit Symbol Substitution Test; EGCG: epigallocatechin-3-gallate; EPA: eicosapentaenoic acid; ERK: extracellular receptor kinase; EVOO: extra-virgin olive oil; FACIT: Folic Acid and Carotid Intima-media Thickness; FFQ: food frequency questionnaire; Gb: *Ginkgo biloba*; GMS-AGECAT: geriatric mental state automated geriatric examination for computer-assisted taxonomy; HCs: healthy controls; HEI: healthy eating index; HEI-2005: Healthy Eating Index-2005; HPA: hypothalamic–pituitary–adrenal; IL: interleukin; HVLT-R: Hopkins Verbal Learning Test—Revised; IADL: instrumental activities of daily living; JNK: The c-Jun N-terminal kinase; LC: long chain; LDL: low-density lipoprotein; LPS: lipopolysaccharide; MAD: modified Atkins diet; MAPT: Multidomain Alzheimer Preventive Trial; MCI: mild cognitive impairment; MCs: controls with medical illness; MCT: medium-chain triglycerides; MedDiet: Mediterranean diet; MIND Diet: MedDiet-DASH Intervention for Neurodegenerative Delay; MMSE: mini-mental state examination; MMSE-2-EV: MMSE-2-Expanded Version; MRI: magnetic resonance imaging; fMRI: functional MRI; mRNA: messenger ribonucleic acid; mTOR: mammalian target of rapamycin; MUFAs: monounsaturated fatty acids; NCDs: non-communicable diseases; NF-kB: nuclear factor kappa B; NHANES: National Health and Nutrition Survey; NIA: National Institute on Aging; NILS-LSA: National Institute for Longevity Sciences, Longitudinal Study of Aging; NMDA: N-methyl-D-aspartate; NPDP: Nordic Prudent Dietary Pattern; NPI: Neuropsychiatric Inventory; OSA: obstructive sleep apnea; PATH: Personality and Total Health; PEGB: polyphenol-rich extract from grape and blueberry; POMS-BI: Profile of Mood States, bipolar form; PREDIMED: Prevención con Dieta Mediterranea; PUFAs: polyunsaturated fatty acids; RCTs: randomized control trials; REGARDS: Reasons for Geographic and Racial Differences in Stroke; SCFAs: short-chain fatty acids; SHARE: Survey of Health, Ageing and Retirement in Europe; SSP: Spatial Span; SUCCAB: Swinburne University Computerized Cognitive Assessment Battery; TLRs: Toll-like receptors; TMT: Trail-Making Test; TNF: tumor necrosis factor; USA: United States of America; VDR: vitamin D receptor; VFT: Verbal Fluency Test; VRM: verbal episodic and recognition memory; VWMH: volume of white matter hyperintensities; WHO: World Health Organization; WISH: The Women’s Isoflavone Soy Health.
